# Genetic variation in *PTPN1* contributes to metabolic adaptation to high-altitude hypoxia in Tibetan migratory locusts

**DOI:** 10.1038/s41467-018-07529-8

**Published:** 2018-11-26

**Authors:** Ding Ding, Guangjian Liu, Li Hou, Wanying Gui, Bing Chen, Le Kang

**Affiliations:** 1 State Key Laboratory of Integrated Management of Pest Insects and Rodents, Institute of Zoology, Chinese Academy of Sciences, 100101 Beijing, China; 2 University of Chinese Academy of Sciences, Beijing, 100049 China; 3 Novogene Bioinformatics Institute, 100083 Beijing, China

## Abstract

Animal and human highlanders have evolved distinct traits to enhance tissue oxygen delivery and utilization. Unlike vertebrates, insects use their tracheal system for efficient oxygen delivery. However, the genetic basis of insect adaptation to high-altitude hypoxia remains unexplored. Here, we report a potential mechanism of metabolic adaptation of migratory locusts in the Tibetan Plateau, through whole-genome resequencing and functional investigation. A genome-wide scan revealed that the positively selected genes in Tibetan locusts are predominantly involved in carbon and energy metabolism. We observed a notable signal of natural selection in the gene *PTPN1*, which encodes PTP1B, an inhibitor of insulin signaling pathway. We show that a *PTPN1* coding mutation regulates the metabolism of Tibetan locusts by mediating insulin signaling activity in response to hypoxia. Overall, our findings provide evidence for the high-altitude hypoxia adaptation of insects at the genomic level and explore a potential regulatory mechanism underlying the evolved metabolic homeostasis.

## Abstract (web summary)

Vertebrate adaptation to high-altitude life has been extensively investigated, while invertebrates are less well-studied. Here, the authors find signals of adaptive evolution in genomes of migratory locusts from the Tibetan Plateau, and implicate a *PTPN1* coding mutation in their hypoxia response.

## Introduction

Animals, including humans, have evolved both physiological and genetic adaptation to low-oxygen availability in high-altitude regions in their range of expansion. For example, the Tibetan Plateau has an average altitude of approximately 4000 m and represents one of the most extreme environments inhabited by humans and numerous animals. Many wild animals and indigenous human populations, as well as their domesticated animals, have highly adapted to life with hypobaric hypoxia. Revealing the mechanisms underlying organismal adaptation to high-altitude hypoxia is attracting considerable attention and can contribute to our understanding of hypoxia-featured human diseases, such as heart failure and various cancers.

Enhancing tissue oxygen uptake and delivery is crucial to such adaptation to lifelong hypoxia. For example, Tibetans can maintain a normal hemoglobin concentration but have a higher level of blood flow than lowlanders, whereas Andean natives have high hemoglobin concentration^1^. Meanwhile, yaks in the Tibetan Plateau and deer mice in the Rocky Mountains have hemoglobin with high-oxygen affinity^2^. Thus hemoglobin is undergoing natural selection in various organisms and is associated with enhanced oxygen delivery^3–6^. Genetic studies on human and other mammals based on whole-genome sequencing revealed strong selection on hypoxia-inducible factor (HIF) pathway-associated genes, such as *EPAS1* and *EGLN1*, which are involved in the regulation of hemoglobin concentration and angiogenesis^7–9^.

Adaptation to high-altitude hypoxia involves mechanisms other than enhanced oxygen delivery. Several studies have emphasized the central role of metabolic changes in hypoxia adaptation. For example, Himalayan Sherpas demonstrate metabolic adaptation at high altitude. Compared with lowlanders, Sherpas exhibit improved tricarboxylic acid (TCA) cycle and oxidative phosphorylation, as well as reduced fatty acid oxidation in the skeletal muscles^10^. Genetic variant analysis has also implicated the involvement of metabolic pathways in the adaptive processes, such as those in yak^11^, Tibetan antelope^12^, Tibetan ground tit^13^, and some domesticated animals^14,15^. However, the genetic mechanisms behind the adaptive metabolic changes remain unclear.

**Fig. 1:**
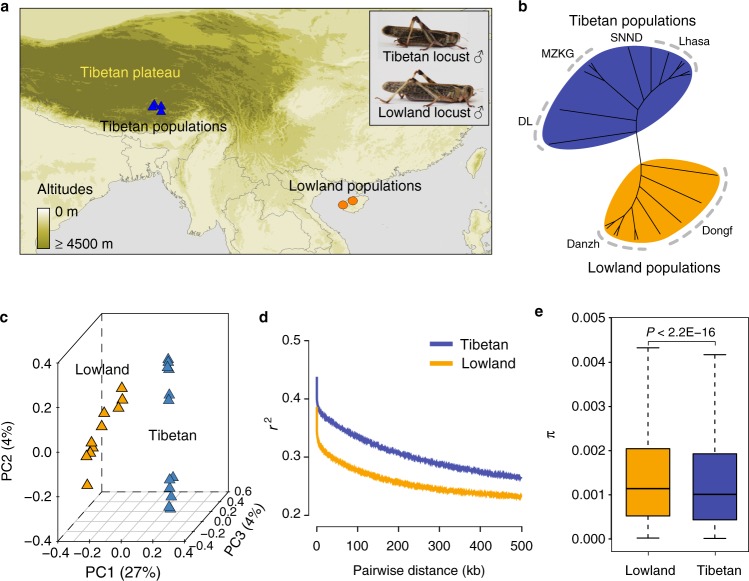
Phylogenetics of migratory locust based on whole-genome SNPs. **a** Geographic distribution of collected locust samples used for whole-genome re-sequencing. Tibetan and lowland locusts are shown at the top-right corner. **b** Unrooted neighbor-joining tree of migratory locust. **c** Principle component (PC) analysis. **d** Patterns of LD (linkage disequilibrium) decay across the genome in different geographic populations. *r*^2^, Pearson’s correlation coefficient. **e** Significant difference in population nucleotide diversity (*π*), as evaluated using the Wilcoxon rank-sum test (*P* < 2.2E−16). The boxplot represents mean value and variance. Maps were generated using DIVA-GIS (http://www.diva-gis.org/). Photographs of locusts were taken by D.D.

Animals with wide altitudinal distribution represent a promising system for unveiling the genetic basis of hypoxia adaptation. However, related studies mainly involve vertebrates rather than invertebrates. The insect tracheal system, with a completely different gas exchange system, transports oxygen directly into tissues. Under artificial hypoxia, insects exhibit a series of adaptive features, such as decreased body size, extended tracheal terminals, enhanced hypoxia resistance, and genetic changes^16–19^. The migratory locust *Locusta migratoria* is one of the most widely distributed pests in the Old World and has inflicted disastrous damage to agriculture. The Tibetan locust populations colonized the Tibetan Plateau over 34,000 years ago and can thrive at 4200 m in altitude^20^. Tibetan locust populations have evolved distinct morphological and physiological traits that render them superiorly able to cope with the chronic shortage of ambient oxygen. Tibetan locusts have much stronger hypoxia tolerance and smaller body size than conspecific lowland locusts (Fig. 1a). Comparative analysis reveals that Tibetan locusts efficiently utilize aerobic metabolism in response to extreme hypoxia^21^. Tibetan locusts' musculature displays a well-maintained mitochondrial structure and enhanced cytochrome c oxidase activity that endow them with excellent long flight and locomotor abilities under hypoxia^22^. Thus Tibetan migratory locusts are an ideal model to study the mechanisms of adaptation to high-altitude hypoxia, although the genetic mechanism underlying such adaptation remains unexplored.

In the current study, we performed whole-genome resequencing and transcriptional analysis of Tibetan and lowland locust populations, as well as functional investigations of adaptive metabolic changes. Genetic analysis revealed positive selection of genes enriched in energy metabolism pathways, and the adaptive response of these pathways to hypoxia was corroborated by transcriptional analysis. A *PTPN1* coding variant shows strong signals of differentiation between altitudes. *PTPN1* encodes tyrosine-protein phosphatase non-receptor type 1 (PTP1B), an inhibitor of insulin receptor. The coding variant facilitates maintaining of homeostasis of carbohydrate and energy metabolism under hypoxia by regulating the activity of the insulin/insulin-like growth peptide signaling (IIS) pathway in Tibetan locusts. Our findings reveal a specific mechanism for metabolic adaptation to high-altitude hypoxia by insects and improve the understanding of the complex biological features of high-altitude adaptation in animals.

## Results

### Genomic differentiation and demographic history

We performed whole-genome resequencing of 22 individuals representing two geographically distinct migratory locust populations in China; 12 individuals were from the Lhasa, Doilung, Shannan, and Maizhikungga localities in the Tibetan Plateau, and 10 were from the Dongfang (Dongf) and Danzhou (Danzh) localities in Southern lowland (Fig. 1a and Supplementary Table 1). The Tibetan locusts were approximately 20% smaller in body size than lowland locusts, although they were taxonomically the same species. The divergence in body size between the Tibetan and lowland locusts persisted even after two generations’ rearing under normoxic condition in the laboratory (Supplementary Fig. 1).

A total of 1675 Gbp of high-quality data were uniquely mapped to the 6.5 Gbp locust reference genome^23^. The average sequencing depth of each individual was at least 10× (Supplementary Table 2 and Supplementary Fig. 2). After applying quality-control criteria, we identified a total of 12 M high-quality single-nucleotide polymorphisms (SNPs). Our analysis was based on these whole-genome SNP variations.

The neighbor-joining (NJ) tree of the locusts featured two distinct clusters (Fig. 1b). The Tibetan and lowland geographical populations formed two branches and were distinctly divided from each other. These groupings were also supported by principal component analysis (PCA; Fig. 1c). In PCA, the first PC explained 27.34% of the total variation that separated the Tibetan and lowland populations. Genetic structure analysis demonstrated a clear division between Tibetan and lowland populations at *K* = 2. At *K* = 3, Lhasa and non-Lhasa populations in Tibetan locusts were divided. At *K* = 4, Dongf and Danzh populations in lowland locusts were separated (Supplementary Fig. 3). Elevated genome-wide linkage disequilibrium (LD) (Fig. 1d) and homozygosity level (Supplementary Fig. 4) but reduced genetic diversity *π* (Fig. 1e) were observed in the Tibetan populations.

To reconstruct the demographic history of the Tibetan and lowland locusts, we initially used the pairwise sequentially Markovian coalescent (SMC) method^24^. Analysis results showed that the effective population size (*N*_e_) of the Tibetan and lowland locusts were highly correlated 90,000 years ago. A peak of the *N*_e_ at 1 million years ago and a constant reduction since then was observed. The *N*_e_ of lowland locusts appeared to be less affected by last glacial period than the Tibetan locusts. In the last glacial maximum (LGM), the *N*_e_ of the Tibetan locusts reached the lowest level and recovered slightly after LGM (Supplementary Fig. 5a). We then performed multiple SMC (MSMC) approach to evaluate divergence time^25^. The curve of relative cross coalescence rates (CCRs) was 0.5 at approximately 90,000 years ago and reached maximum values at about 1 million years ago. The curve converged to zero for recent times, indicating hardly any gene flow between the two populations (Supplementary Fig. 5b). Thus we estimated that the two populations split at approximately 90,000 years ago.

### Evidence for metabolic adaptation in Tibetan locusts

**Fig. 2:**
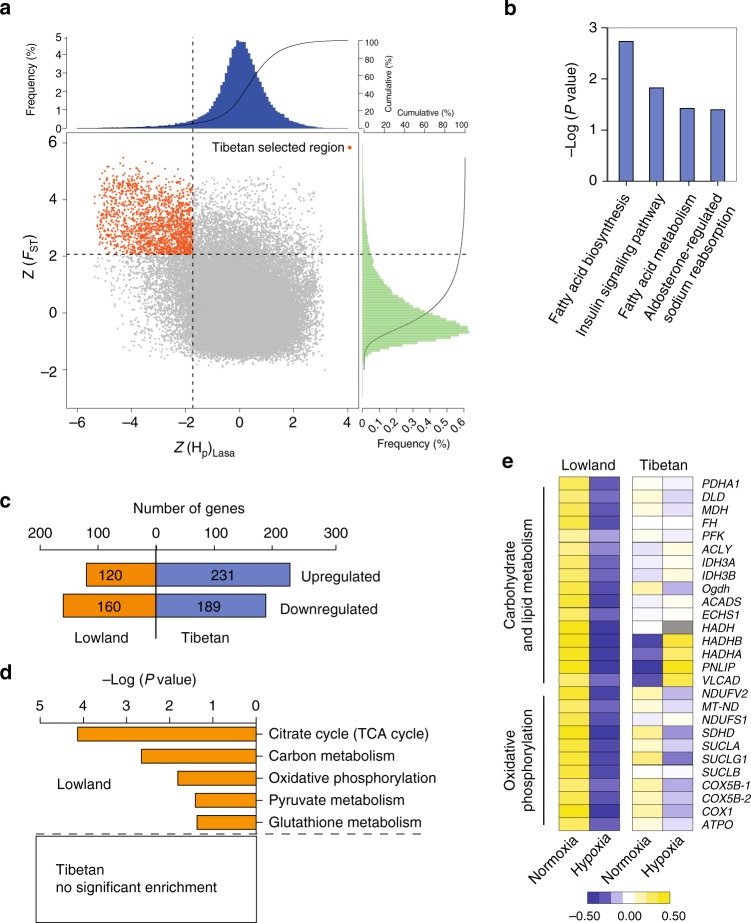
Selective sweep and expression analysis of hypoxia adaptation. **a** Distribution of *Z*-transformed fixation (Z*F*_ST_) and heterozygosity (Z*H*_P_), calculated in 100-kb sliding windows. Genomic region (red point) located at top left of the dash lines (5% of empirical maximum *F*_ST_ and minimum *H*_P_) is identified as high-altitude adaptation-associated region in Tibetan locusts. Genes in this region are positively selected genes (PSGs). **b** KEGG enrichment of PSGs. **c** Number of differentially expressed genes (DEGs) in response to hypoxia induction through transcriptome sequencing. **d** KEGG enrichment of DEGs. Only KEGG terms with *P* < 0.05 are shown (Fisher’s Exact Test). **e** Expression pattern of DEGs involved in energy metabolism. Heat map signal indicates log2 fold-change values relative to the median expression level within the group. Yellow signal represents higher expression and blue represents lower expression relative to the median level within the group

To identify the genomic region that present signatures of natural selection associated with high altitude, we performed whole-genome genetic differentiation analysis between Tibetan and lowland locusts. Using 5% maximum *Z*-transformed fixation (Z*F*_ST_) and 5% minimum pooled heterozygosity (Z*H*_P_), we identified a total of 113.8 Mb genomic regions that covered all outliers (Fig. 2a). These regions contained 484 annotated positively selected gene (PSG) candidates (Fig. 2a). Among these PSGs, 137 showed amino acid substitution. Kyoto Encyclopedia of Genes and Genomes (KEGG) signaling pathway analysis demonstrated that fatty acid biosynthesis (*P* = 1.9 × 10^−3^, Fisher’s Exact Test), insulin signaling pathway (*P* = 0.015), fatty acid metabolism (*P* = 0.038), and aldosterone-regulated sodium reabsorption pathway (which is closely related to carbohydrate digestion and absorption, *P* = 0.040) were the most highly enriched (Fig. 2b). These pathways are all related to metabolic regulation. Gene Ontology (GO) analysis of PSGs demonstrated high enrichment in the GO terms, namely, cofactor metabolic process (*P* = 0.015) and oxidoreduction coenzyme metabolic process (*P* = 0.030), which are involved in energy metabolism (Supplementary Table 3).

To determine whether the enriched metabolic regulatory pathways account for hypoxia adaptation, we conducted transcriptomic analysis of the thoracic muscle of Tibetan and lowland locusts treated with 10% partial oxygen pressure (*P*o_2_) hypoxia. The thoracic muscle of adults was subjected to the analysis because this tissue was actively involved in anaerobic and aerobic metabolism (Supplementary Fig. 6). Gene expression levels under normoxic and hypoxic conditions were compared. Interestingly, we identified more differentially expressed genes (DEGs) in Tibetan locusts than in lowland locusts (420 vs. 280, respectively). More genes were upregulated than downregulated in Tibetan locusts (231 vs. 189, respectively), a pattern in contrast to that observed in the lowland locusts (120 vs. 160, respectively) (Fig. 2c). KEGG analysis demonstrated that the DEGs were highly enriched in lowland locusts’ pathways related to carbohydrate and energy metabolism, i.e., citrate cycle (*P* = 7.3 × 10^−5^, Fisher’s Exact Test), carbon metabolism (*P* = 2.2 × 10^−3^), oxidative phosphorylation (*P* = 0.015), and pyruvate metabolism (*P* = 0.039). Conversely, no significant enrichment of KEGG pathways was observed in Tibetan locusts (Fig. 2d). The DEGs involved in energy metabolism showed overall suppressed expression after hypoxia induction in lowland locusts but were stably expressed in Tibetan locusts (Fig. 2e). The differential expression of glycolysis-related genes in response to hypoxia between the two locust populations was further validated by quantitative PCR (qPCR) (Supplementary Fig. 7). GO analysis also exhibited a similar pattern in metabolic processes (Supplementary Fig. 8). These results indicate that energy metabolism in lowland locusts is highly repressed by hypoxia, whereas Tibetan locusts evolved metabolic robustness against hypoxic stress.

Therefore, both whole-genome genetic and expression analysis pointed to the involvement of energy metabolic pathways in hypoxia adaptation. In addition to the genes involved in metabolism, the top 20 genes with the highest Z*F*_ST_, cross-population composite likelihood ratio (XP-CLR), or *∆*Z*H*p value were examined. Genes involved in tracheal growth (*if* and *Aggf1*), wing and muscle development (*osa*, *su(dx)*, *CAP1*, and *Klhdc1*), cell differentiation and division (*Efhc1*, *D1Pas1*, *Gas2l1*, and *Ctdspl2*), and oxidative stress response (*stau2*) were likely involved in the adaptation to hypoxia or high altitude in Tibetan locusts (Supplementary Tables 4–6).

### Positive selection of the *PTPN1* variants

**Fig. 3:**
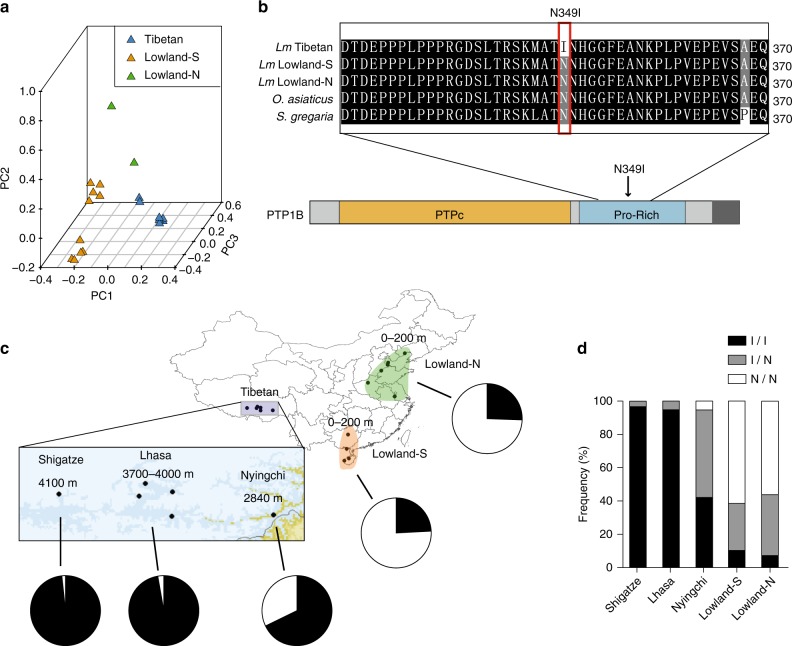
Genetic differentiation of *PTPN1*. **a** Principle component analysis plot of geographically different locust populations based on the SNPs of the *PTPN1* gene region. Lowland-S and Lowland-N represent South and North China lowland locust populations, respectively. **b** Multi-species alignment of amino acid sequence of PTP1B encoded by *PTPN1*. *Oedaleus asiaticus* and *Schistocerca gregaria* are two outgroup species closely related to *Locusta migratoria* (abbreviated as *Lm*). The missense mutation p.Asn349Ile (N349I) is located at the proline-rich (Pro-Rich) domain of PTP1B. **c** Mutation frequency of p.Asn349Ile in Tibetan and lowland locusts. The pie chart shows the proportion of p.Asn349Ile allele frequency in each population. Sample size: *n* ≥ 38. **d** Allele frequency of mutant loci in PTP1B. I/I, I/N, and N/N represent homozygous wild type, heterozygote mutant, and homozygous mutant for p.Asn349Ile, respectively. Maps were generated using DIVA-GIS (http://www.diva-gis.org/)

To identify the major genetic loci responsible for metabolic adaptation in Tibetan locusts, we analyzed the 13 PSGs that were enriched in energy metabolism-related pathways, namely, *PTPN1*, *PIK3CD*, *FAS1*, *FAS2*, *FAS3*, *ACO1*, *FAR1*, *SDHD1*, *Ndufb7*, *ADIPOR*, *ATP1A2*, *VLCAD*, and *PPT1*. Among these PSGs, only *PTPN1*, *FAS1*, *FAS3*, *ATP1A2*, and *PPT1* had nonsynonymous mutations (Supplementary Table 7). *PTPN1* locus showed a high XP-CLR score, a high average LD (*r*^2^ = 0.8) and a low Tajima’s *D* (Supplementary Fig. 9). These results suggest that *PTPN1* is under strong positive selection at high altitude. To further confirm the association of *PTPN1* variants with altitude but not with latitude, we performed whole-genome resequencing of two additional locusts from North China lowlands (Supplementary Tables 1 and 2). PCA demonstrated that *PTPN1* variants in Tibetan locusts were separated from the locusts in both South and North China lowlands (Fig. 3a).

*PTPN1* encodes the protein PTP1B, which is conserved among insects and humans, especially at the protein tyrosine phosphatase (PTP) domain (where 61% similarity was observed between that of locusts and humans) (Supplementary Fig. 10). High-throughput sequencing and Sanger sequencing revealed only one nonsynonymous mutation (c.1046A>T) in *PTPN1* in Tibetan locusts, which encodes the amino acid substitution p.Asn349Ile at the proline (Pro)-rich domain of PTP1B (Fig. 3b). The mutation frequency in Tibetan locusts at >3700 m altitudes (Lhasa 3700–4000 m and Shigatze 4100 m) was >98%, whereas that in Ningchi (2840 m), South China lowlands and North China lowlands dropped to 68%, 24%, and 26%, respectively (Fig. 3c). Furthermore, The *PTPN1* point mutant in Tibetan locusts at the altitudes of >3700 m showed higher homozygosity than all the other populations (Fig. 3d). The results indicate that the mutation in *PTPN1* is under strong directional selection for altitude adaptation in the locust.

### *PTPN1* mutation attenuates IIS pathway suppression in hypoxia

**Fig. 4:**
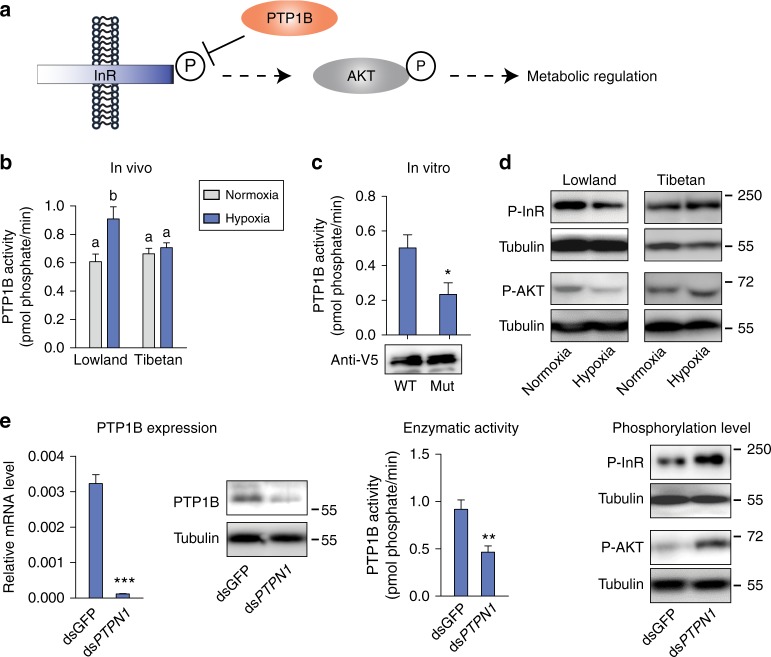
Effect of *PTPN1* mutation on the insulin signaling pathway. **a** Illustration of the insulin signaling pathway, in which PTP1B is a negative regulator of the pathway through the dephosphorylation of insulin receptor at its tyrosine site. **b** PTP1B enzyme activity differing between Tibetan and lowland locusts in response to hypoxia induction. Significant differences are denoted by different letters (one-way ANOVA, *P* < 0.05) (*n* = 6 replicates). The values of the columns here and below are shown as mean ± standard error (s.e.m.). **c** The coding mutation altered PTP1B enzyme activity in vitro. The wild-type p.Asn349 (WT) and mutant-type p.Ile349 (Mut) PTP1B were overexpressed in S2 cells and induced by 1% hypoxia for 6 h. PTP1B production level was detected by western blot with anti-V5 tag antibody. *P < 0.05 by Student’s *t* -test. *n* = 3 replicates. Supplementary Fig. 15d shows the original image. **d** Western blot revealed decreased InR and AKT phosphorylation level after hypoxia induction in lowland locusts. P-InR and P-AKT represent phosphorylation of InR and AKT, respectively. The molecular weight markers are shown on the right side of the blot. Supplementary Figs. 15e–h show the original images. **e** PTP1B knockdown abolished PTP1B mRNA and protein level, repressed PTP1B activity, and increased InR and AKT phosphorylation level under hypoxic condition. The levels were examined 8 days after dsRNA injection. ds*PTPN1* represents *PTPN1* dsRNA knockdown. ds*GFP* was used as a negative control. PTP1B knockdown assay was performed with the lowland locusts. **P < 0.01, ***P < 0.001 by Student’s *t* test. *n* ≥ 3 replicates. Supplementary Figs. 15i–k show the original images

To determine the effect of the missense *PTPN1* mutation on PTP1B enzyme activity, we examined PTP1B activity in vivo. PTP1B negatively regulates the insulin pathway through insulin receptor (InR) dephosphorylation (Fig. 4a). Under normoxic conditions, no difference in PTP1B enzyme activity between Tibetan and lowland locusts was detected. However, PTP1B enzyme activity was elevated by hypoxia induction in lowland locusts but remained stable in Tibetan locusts (Fig. 4b). PTP1B did not respond to hypoxia challenge at both the mRNA and protein levels in lowland and Tibetan locusts (Supplementary Fig. 11a). These results suggest that the alteration of PTP1B activity is due to protein structural change. To examine the causal relationship between the mutation point and enzyme activity of PTP1B, we first conducted an in vitro assay using recombinant PTP1B protein (Supplementary Fig. 11b). The purified recombinant PTP1B showed clear phosphatase activity compared with bovine serum albumin (BSA). However, no significant differences in PTP1B enzyme activity between the wild-type p.Asn349 and mutant-type p.Ile349 were observed (Supplementary Fig. 11c). We then speculated that locust PTP1B activity required hypoxia induction and the presence of cofactors. Thus we overexpressed locust PTP1B in S2 cells and subjected the cells to hypoxia for 6 h. The mutant PTP1B presented a significantly lower enzyme activity than the wild type. The in vitro result was consistent with that observed in vivo, indicating that the high-altitude dominant mutation attenuates the hypoxic response in terms of PTP1B enzyme activity (Fig. 4c).

Afterwards, we tested the phosphorylation level of InR, the substrate that is dephosphorylated by PTP1B, in both lowland and Tibetan locusts. The phosphorylation level of InR was reduced in lowland locusts after hypoxia induction but remained unchanged in Tibetan locusts. Accordingly, the phosphorylation level of the protein kinase B AKT, an important kinase in the insulin pathway, was also suppressed under hypoxic condition in lowland locusts but remained normal in Tibetan locusts (Fig. 4d). During this process, the insulin-like peptide (Ilp) produced in the brain and thoracic muscle did not respond to hypoxia induction in both lowland and Tibetan locusts (Supplementary Fig. 12). These results demonstrate that the inhibitory effects of the PTP1B variant on IIS signaling are not caused by reduced Ilp production but by repressed InR phosphorylation.

Finally, we validated the function of locust PTP1B in InR and AKT phosphorylation regulation by *PTPN1* gene expression knockdown in vivo. RNA interference (RNAi) through double-strand RNA (dsRNA) injection in lowland locusts dramatically reduced the *PTPN1* expression and PTP1B enzyme activity (Student's *t* test, *P* = 0.009). The *PTPN1* knockdown elevated the phosphorylation levels of InR and AKT under hypoxic conditions (Fig. 4e). Thus the *PTPN1* mutation represses InR phosphorylation and downstream insulin signaling pathways in locusts.

### PTP1B regulates metabolic adaptation to hypoxia

**Fig. 5:**
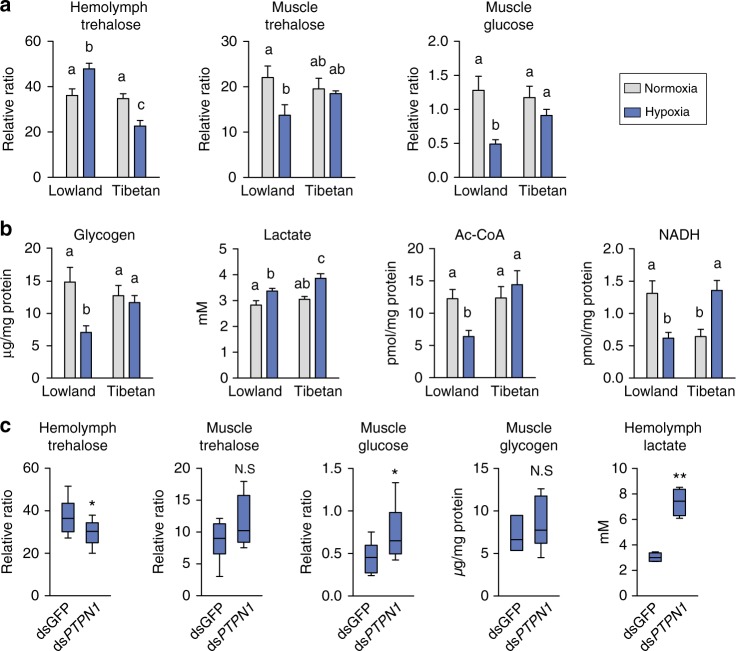
Effect of *PTPN1* mutation on energy metabolism alteration. **a** Trehalose level and glucose concentration in hemolymph and thoracic muscle. The relative ratio is the ratio of the peak area of metabolic intermediate to the peak area of the internal standard (sucrose). (*n* ≥ 6 replicates, 3 locusts/replicate). **b** The levels of glycogen, acetyl-CoA, and NADH in the thoracic muscle and lactate in hemolymph. Values in **a**, **b** are the mean ± s.e.m. Significant differences are denoted by letters (one-way ANOVA, *P* < 0.05) (*n* = 4 replicates, 3 locusts/replicate). **c** PTP1B knockdown affected hypoxia-induced metabolic regulation by the insulin pathway (*n* = 5 replicates, three locusts/replicate). *P < 0.05, **P *<* 0.01 (Student’s *t* test). N.S. represents no significant difference (Student’s *t* test). The center line of the boxplots represents median value, the bounds of the box represent 75th and 25th percentile, and the whiskers represent maximum and minimum value

Finally, we attempted to gain insight into the adaptive changes in energy metabolism regulated by the IIS pathway under high-altitude hypoxia. Considering that insulin is an important sugar-homeostasis regulatory hormone in animals and is involved in energy metabolism control, we first examined trehalose uptake ability by measuring trehalose level in hemolymph and thoracic muscle. Trehalose content was significantly increased in the hemolymph but dropped by 40% in the muscle cells in lowland locusts after hypoxia exposure. Glucose, the hydrolysis product of trehalose, was also dramatically reduced in the muscle cells. By contrast, trehalose in hemolymph was reduced but intracellular trehalose and glucose in the thoracic muscle were maintained at normal levels in Tibetan locusts after hypoxia challenge (Fig. 5a).

Second, we measured the glycogen content after hypoxia challenge because glucose is involved in glycogenesis or glycolysis after entering a muscle cell. Glycogen level dropped by nearly 50% in lowland locusts but did not significantly change in Tibetan locusts. Third, we measured the production of lactate and acetyl coenzyme A (CoA), the product of glycolysis. Lactate level was elevated in both lowland and Tibetan locusts after hypoxia induction; conversely, the acetyl-CoA content was reduced only in lowland locusts. We also measured NADH production, an important energy product in TCA cycle and glycolysis. NADH content was significantly reduced in lowland locusts but was elevated in Tibetan locusts after hypoxia induction (Fig. 5b). Meanwhile, we tested the protection role of trehalose in cell survival under hypoxia tension. In situ cell death detection assay indicated that trehalose injection rescued cells from dying induced by severe hypoxic condition in lowland locusts (Supplementary Fig. 13).

We further investigated the effects of PTP1B on energy metabolism. The expression knockdown of *PTPN1* in lowland locusts significantly reduced the level of hemolymph trehalose, increased the level of intracellular glucose, and dramatically increased lactate production by nearly three times. The results indicate that the capacity of trehalose uptake in lowland locusts under hypoxic condition improved after suppressing PTP1B activity (Fig. 5c).

We finally examined the fitness effect of *PTPN1* knockdown, i.e., the adult lifespan of locusts under hypoxia condition. The dsRNA interference of *PTPN1* caused at least 97% reduction in the mRNA and protein levels of *PTPN1* expression (Supplementary Fig. 14a). Under normoxic condition, *PTPN1* repression had no effect on the lifespan of adult locusts compared with the control. However, upon hypoxic rearing, their lifespan significantly decreased by 5 days after *PTPN1* downregulation (Wilcoxon rank-sum test; *P* = 0.004; Supplementary Fig. 14b). The results indicate that adult locusts living under hypoxia suffers a shortened lifespan upon the repression of *PTPN1* gene expression.

## Discussion

The genetic mechanism for adaptation to high-altitude hypoxia by insects, which employ a tracheal system for oxygen delivery, was investigated in this work. The majority of previous studies on high-altitude adaptation have focused on vertebrates, especially mammals and humans, which use hemoglobin for oxygen transport. Several studies have reported evidence for positive natural selection of genes associated with hemoglobin phenotypes and erythropoiesis. This finding raised an intriguing question as to whether insects have evolved strategies different from those of vertebrates to cope with the lifelong extreme hypoxia environments. We attempted to resolve the question in the migratory locust, which is featured by global distribution and high capacity for adaptation to changing environments, including variation in oxygen levels along altitudes. The Tibetan locusts have evolved distinct high-altitude adaptive features in their long-term habitation in the Tibetan Plateau, particularly the superior resistance to hypoxia^21,22^. Accordingly, we used the Tibetan locust as a model to dissect the genetic basis of its adaptation to high-altitude hypoxia.

In addition to the robust hypoxia resistance and metabolic change of the Tibetan locust, it also exhibits a dramatic reduction in body size that is heritable. Many studies have revealed a close relation between insect body size and ambient oxygen content. In the late Paleozoic when the atmospheric *P*_O2_ was hyperoxic (>30%), the insects were 10 times larger than those alive today^26^. When reared under an artificial hypoxic condition, some insects such as *Drosophila melanogaster* and *Manduca sexta* showed a significantly reduced body size^27,28^. These findings are consistent with the hypothesis that body size is regulated by a mechanism that senses oxygen limitation. The decrease in body size may be adaptive for insects living in hypoxia, because oxygen diffusion distances are shortened, metabolic expenditure is reduced, and flight locomotor energetics are promoted^26^. However, artificial hypoxia did not affect the final body size of *Schistocerca Americana*^17^. By contrast, the bumble bee (*Bombus*) and *Drosophila* inhabiting high altitudes exhibit larger body sizes than their lowland counterparts^29,30^. The body size development of insects is regulated by a number of ecological factors such as temperature, nutrition, gut flora, and infection in addition to oxygen supply^31^. Tradeoffs between the response to hypoxia and other factors may constrain body size evolution. Some biotic interactions, such as predation and competition, may also supersede oxygen as the most important constraint on the body size of insects^32^.

Laboratory selection in the fruit fly has demonstrated that the evolution of hypoxia tolerance involves altered gene expression and genetic codes^18,28^. However, a genomic analysis of honey bees at different altitudes (1100–2900 m) in East Africa reveals nearly no genetic differentiation between lowland and highland populations, although two extended haplotype blocks harboring several octopamine receptor genes are highly diverged between the two altitudes^33^. Our genomic variation analysis based on 12 M SNPs revealed a clear differentiation in genetic background between the Tibetan and lowland locusts, although these populations belong to the same lineage based on mitochondrial genomes^20^. Tibetan locusts also presented lower levels of nucleotide diversity and haplotype homozygosity than lowland locusts. These variations may represent directional selection and provide a rich source of candidate selected loci that may involve in the high-altitude adaptation of the insects.

Genetic and gene expression analysis in the locusts recapitulated the evolutionary conservation of metabolic adaptation of insects, animals, and even humans living in sustained hypoxia. Among the PSGs of Tibetan locusts, many were highly enriched in energy metabolism pathways. Transcriptomic analysis demonstrated that almost all genes in carbon and energy metabolism pathways were significantly repressed by hypoxia stress in lowland locusts. By contrast, Tibetan locusts exhibited a metabolic homeostasis in gene expression. These findings are consistent with previous ones from microarray data of lowland locusts^34^. More genes were upregulated by hypoxia in Tibetan locusts than in lowland locusts (Fig. 2c), implying a wide array of adaptive mechanisms evolved in Tibetan locusts to cope with stress. Wild locusts highly depend on energy supplement for their daily activities (e.g., mating, foraging, or predator avoiding), thereby posing a high demand for cellular metabolic homeostasis under chronic hypoxia. Himalayan Sherpas also exhibit enhanced oxygen utilization efficiency and improved muscle energetics under hypoxia^10^. A genetics study on Tibetan ground tits has shown an expansion in genes linked to energy metabolism^13^. Therefore, metabolic adaptation is a crucial and conserved component of adaptive features of high-altitude animals.

The genetic analysis of candidate genes involved in carbohydrate metabolism demonstrated that *PTPN1* was under strong positive selection and had high divergence at a genetic level between Tibetan and lowland locusts. PTP1B, the protein encoded by *PTPN1*, contained a missense mutation p.Asn349Ile in the proline-rich (Pro-rich) domain in Tibetan locusts. The mutation frequency ranging between 26% and 98% is correlated with altitude elevation at 20–4100 m. PTP1B belongs to the PTP superfamily and is known as a negative regulator of the insulin pathway through the dephosphorylation of InR and its substrate insulin receptor substrate^35^. Thus PTP1B is used as a target of drugs for insulin resistance and type-2 diabetes. PTP1B is overexpressed in one-third of human breast cancer patients^36^ and is essential for tumor survival during hypoxia by regulating non-mitochondrial oxygen consumption^37^. *PTP61F* (the *Drosophila* ortholog of *PTPN1*) is responsible for the reduced InR activity under hypoxic condition in S2 cells^38^. An evolutionary genomic study of Tibetan wild boar has also revealed that *PTPN1* is in the list of high-altitude adaptation-related genes^39^. The lifespan cost resulting from *PTPN1* downregulation in a hypoxia environment recapitulates the significant role of this gene in the hypoxia adaptation of the locust. *PTPN1* repression induces high IIS activity and energy metabolism, which may cause excessive accumulation of oxidative stress, as shown in *Drosophila* and *Caenorhabditis*^28,40^. Thus, in addition to cellular hypoxia regulation, PTP1B can be crucial to high-altitude hypoxia adaptation.

Our functional studies confirmed that a specific missense mutation in PTP1B altered its enzyme activity of dephosphorylation and insulin pathway signaling regulation in response to hypoxia induction. Hypoxia exposure in lowland locusts caused elevated PTP1B enzyme activity, which led to suppressed insulin pathway and downstream energy metabolism. PTP1B activity in Tibetan locust remained stable upon hypoxia induction. The reduction-of-function mutation of PTP1B facilitated the maintenance of the IIS pathway and energy metabolism in Tibetan locusts under hypoxic condition. In vivo and in vitro experiments confirmed the signaling and metabolic consequences of the mutation in PTP1B. The point mutation of PTP1B is located in the Pro-rich domain, which is a regulatory domain of PTP1B. Four mechanisms are known to regulate PTP1B activity: oxidation at the N-terminal catalytic phosphatase domain, phosphorylation at both catalytic and Pro-rich domain, sumoylation at the Pro-rich domain, and calpain-mediated proteolysis at the C-terminal near the Pro-rich domain^41^. Thus the missense mutation possibly affects the hypoxia-induced PTP1B modification level and attenuates its enzyme activity alteration. Meanwhile, PTP1B expression interference is consequential for the locust’s fitness (e.g., lifespan). Speculatively, this specific mutation has been selected for optimum hypoxia adaptation and survival under high-altitude environments. Further studies are needed to clarify the molecular mechanism of this process.

The current study revealed a role of the IIS pathway in the evolution of high-altitude hypoxia adaptation through metabolic regulation. The IIS pathway is an evolutionarily conserved nutrient-sensing pathway that modulates energy metabolism and development in metazoans^42^. The reduced activity of IIS can lead to the systematic suppression of energy metabolism in fruit fly^43^, and this phenotype is similar to the hypoxia-induced alteration of energy metabolism in lowland locusts. Previous studies have also provided evidence of the involvement of the IIS pathway in hypoxia adaptation. Reduction-of-function mutations in the *daf-2* gene (encoding an insulin/insulin-like growth factor receptor homolog) can provide powerful protection from hypoxic injury in *Caenorhabditis elegans*^40^. The IIS pathway is closely related to growth and tracheal plasticity under hypoxic conditions in *Drosophila*^44,45^. Insulin can activate HIF-dependent transcription in both S2 cells and *Drosophila* embryos^46^. AKT/phosphoinositide-3 kinase signaling in the downstream of the IIS pathway in the skeletal muscle of mice can respond to acute hypoxia^47^. At the genetic level, altitude-related selection loci in speckled teal (*Anas flavirostris*) also contain genes involved in the IIS^48^, which suggests a conserved regulatory mechanism by IIS underlying the high-altitude hypoxia adaptation. The current study further demonstrated the role of the IIS pathway in energy homeostasis regulation during hypoxia adaptation.

We report a model of hypoxia-induced insulin resistance in locusts. Insulin resistance is an abnormal physiological state that occurs when insulin cannot trigger signaling pathways in target organs, such as the liver, adipose tissues, and muscles. Such physiological models were also established in *Drosophila* and *C. elegans* but were achieved by gene knockout or diet induction^49,50^. Hypoxia can inhibit insulin signaling and induce insulin resistance in adipose tissues originating from obese human or murine^51^, although some studies have reported that high altitude is not a risk factor for insulin resistance-induced type-2 diabetes mellitus in humans^52^. Thus locusts are a potential model for insulin resistance-related obesity studies. Meanwhile, previous work indicates that trehalose can work as a chaperone and prevent the misfolding of substrate proteins^53,54^. Our results indeed demonstrated that elevated intracellular trehalose can rescue muscle cells from death under severe hypoxia condition. Thus, in addition to energy metabolism regulation, the maintained IIS pathway in Tibetan locusts can contribute to severe hypoxia tolerance by stimulating trehalose uptake, which in turn protects cells from hypoxia-induced injury. In summary, these findings may help elucidate hypoxia-induced metabolism syndrome in humans.

Adaptation to high-altitude hypoxia requires multiple systems, pathways, and molecular mechanisms^55,56^. Herein we also identified other PSGs in multiple pathways, such as tracheal growth and wing and muscle development. Tibetan locusts have thrived in the complex habitats on the Tibetan plateau for tens of thousands of years. We speculate that Tibetan locusts have evolved a good balance between energy metabolism and other adaptive traits (e.g., oxidative-stress prevention). Further studies are needed to improve our comprehension of high-altitude adaptation by insects.

## Methods

### Locust sampling and maintenance in the laboratory

We sampled 24 migratory locusts for whole-genome re-sequencing, including 12 locusts from four localities in Tibetan plateau ranging between 3700 and 4000 m in altitude, 10 locusts from two localities in lowland region (<200 m) in Southern China and 2 locusts from lowland region in Northern China. For Sanger sequencing, we sampled 207 migratory locusts, including 127 from 6 localities in Tibetan plateau, ranging between 2840 and 4100 m in altitude, 39 from four lowland localities (<200 m) in Southern China, and 41 from five lowland localities in Northern China (Figs. 1a and  4c and Supplementary Table 1). For the functional study, field-collected locusts were maintained in the laboratory at the Institute of Zoology, Chinese Academy of Sciences in Beijing (<50 m). All locusts were reared in ventilated cages (50 × 50 × 50 cm) at a density of approximately 150 individuals per cage and fed with fresh wheat seedlings and wheat bran. The culturing environment was kept constant with a 14 h light (L):10 h dark (D) photo regime at 28 ± 1 °C. The cultures were maintained in the laboratory for at least two generations prior to the experiments. The 7-day-old male adults were collected for assays.

### Locust body size measurement

The body sizes of locusts were measured with a Vernier caliper. Field-collected and laboratory-reared locust samples were selected randomly for the measurement. Body length was measured from head to wing tip. Wing length was measured from wing root to tip. Femur length is the length of posterior femur. The laboratory-reared locusts were the field-collected locusts reared in the laboratory for more than two generations. The sample numbers for each measurement: field-collected Tibetan male/female, *n* = 8/10; laboratory-reared Tibetan male/female, *n* = 10/10; field-collected lowland male/female, *n* = 12/12; laboratory-reared lowland male/female, *n* = 10/10.

### Genome sequencing and quality control

A total of 1.5 μg genomic DNA per sample was extracted and used as input material for the DNA sample preparations. Sequencing libraries were generated using the Truseq Nano DNA HT Sample Preparation Kit (Illumina, USA) following the manufacturer’s recommendations. Index codes were added to attribute sequences to each sample. DNA was then sheared into fragments of 150 bp and sequenced using the Illumina Hiseq X Ten platform. In total, whole genomes of the 24 locusts were sequenced at an average depth of exceeding 10× using a sequencing strategy similar to that applied in the 1000 Genomes Project (see http://www.1000genomes.org; The1000 Genomes Project Consortium 2010). To obtain reliable reads and avoid filter reads with artificial biases that may affect downstream mapping and other analyses, we implemented quality-control procedures to remove the following types of reads: (1) ≥10% unidentified nucleotides (i.e., N content); (2) >10% with >10 nucleotides aligned to the adaptor or mismatches; (3) >50% of the read bases with a Phred quality score (i.e., *Q*-score) <5, and (4) Putative PCR duplicates generated in the library construction process. Consequently, we generated 1675.19 Gb of high-quality paired-end reads for the 24 locusts with the quality of ≥94.73% of the bases for Q20 and ≥88.44% for Q30. The detailed information of high-quality data for each sample is summarised in Supplementary Table 2.

### Reads mapping and SNP calling

The filtered high-quality reads were mapped to the locust reference genome using Burrows–Wheeler Aligner with the command ‘mem -t 4 -k 32 –M’^57^. Unmapped and secondary alignment reads were filtered and then sorted by SAMtools (Version:1.3.1). Potential PCR duplicates were also removed using ‘rmdup’ command in the SAMtools software. After alignment, we performed SNP calling on a population scale through SAM tools v0.1.30^58^. The command “mpileup“ was used to identify SNPs and indels with the parameters set as ‘-q 1 -C 50 -S -D -m 2 -F 0.002’. To exclude SNP calling errors caused by incorrect mapping or machine error, only high-quality SNPs (coverage depth ≥3 and ≤50, root mean square mapping quality ≥20, maf ≥0.05, miss ≤0.1) were kept for subsequent analysis. Finally, SNP variations were annotated using the ANNOVAR software^59^. Genome coverage rate for each sample was calculated by SAM tools. The saturation curve was generated by calculating the coverage rate of a Tibetan sample (sample ID: Tibetan-5) at sequencing depths of 2, 4, 6, 8, and 10 times.

### Phylogenetic tree and PCA

To clarify the phylogenetic relationship from a genome-wide perspective, we constructed an individual-based NJ tree based on the *p*-distance using the software TreeBestv1.9.2 (http://treesoft.sourceforge.net/treebest.shtml). PCA analysis of the 24 samples was conducted using EIGENSOFT3.0 and the significance of eigenvectors was determined using the Tracey-Widom test in the EIGENSOFT 3.0^60^.

### Population structure

The population genetic structure was examined via an expectation maximization algorithm, as implemented in the program FRAPPEv1.1, which employs the maximum likelihood and expectation-maximization algorithm to estimate ancestry proportions for each individual^61^. The number of assumed genetic clusters *K* ranged from 2 to 4, with 10,000 iterations for each run.

### Demographic history

Changes in *N*_e_ of each group was inferred using a hidden Markov model approach as implemented in pairwise sequentially Markovian coalescence with parameter as follows: −N30 −t15 −r5 and −p ‘4+25*2+4+6’. Time was measured in units of 2*N*_0_ generations, and the *N*_e_ at time *t* was scaled to *N*_0_. The neutral mutation rate *µ* was used to infer *N*_0_ and scale the TMRCA (time to the most recent common ancestor) and *N*_e_ values into chronological time. The mean generation time *g* was set at 0.5 year, and *µ* was estimated as 0.1 × 10^–8^^24^. CCRs for the divergence of Tibetan and lowland populations were estimated under the MSMC model based on four representative samples of each group (Tibetan 6, 7, 8, and 9; and HaiN 1, 2, 4, and 5)^25^.

### Genetic diversity and LD

Nucleotide diversity (*π*) was calculated using the list of high-quality SNPs for each group^62^. To estimate and compare the pattern of LD for different groups, we computed the squared correlation coefficient (*r*^2^) between pairwise SNPs using the software Haplo View v4.2^63^. Parameters in the program were set as ‘-n –dprime-minMAF 0.05’. The average *r*^2^ value was calculated for pairwise markers in a 500-kb window and averaged across the whole genome. Tajima’s *D* values were calculated using VCFTOOLS with the parameter ‘–TajimaD’.

### Selective sweep

To identify genome-wide selective sweeps associated with high-altitude adaptation, we calculated the average pooled heterozygosity (*H*_P_) and the genome-wide distribution of fixation index (*F*_ST_) using a sliding-window approach, which involved 100 kb windows with 50 kb increments. At each detected SNP position, we counted the number of reads corresponding to the most and least frequently observed allele (nMAJ and nMIN, respectively) in each group. The *H*_P_ for each window was calculated based on the equation *H*_P_ = 2∑nMAJ × ∑nMIN (∑nMAJ + ∑nMIN)^−1^ × 2^–1^. *H*_P_ and *F*_ST_ were *Z*-transformed to obtain Z*H*_P_ and Z*F*_ST_. We considered the windows with the top 5% Z*F*_ST_ and Z*H*_P_ simultaneously as candidate outliers under strong selective sweeps. All outlier windows were assigned to corresponding SNPs and genes^64^.

### Cross-population composite likelihood approach

The XP-CLR was used to perform a genome scanning for selective signals^65^. A 0.005-cM sliding window with maximum 200 SNPs assayed was used for scanning. Genetic map of individual SNPs used here was 1 cM Mb^−1^. The command line was XPCLR -xpclr genofile1 genofile2 mapfile outputFile -w1 0.005 200 2000 chrN -p 0.95. Finally, the mean likelihood score was calculated in 50-kb sliding windows with a step size of 25 kb across the genome. The highest XP-CLR values, accounting for 0.5% of the genome, were considered as selected regions.

### Hypoxic treatment

Hypoxic treatment was performed in a hypoxic chamber (FLYDWC-50; Fenglei Co., Ltd, China) in which the ambient temperature and air flow is in automatic control. Twenty male locusts were placed in a cage (20 × 15 × 15 cm) kept in the chamber, into which air was blown and balanced with pure nitrogen to achieve the required *P*o_2_ levels. The *P*o_2_ we used in functional studies was 10 kPa, which is sufficiently induced behavioral, developmental, and metabolic alterations in insects^17^. The locusts were maintained in the chamber for 5 days at 30 ± 1 °C with a constant light cycle (12 L:12 D).

### RNA sequencing and data processing

The thoracic muscle of Tibetan and lowland locusts was collected 5 days after hypoxia and normoxia treatment. The normoxia treatment groups were used as controls. The thoracic muscle of three independent replicates of three locust adults was collected for tissue preparation. Total RNA was extracted by using TRIzol reagent (Invitrogen, USA). cDNA libraries were prepared in accordance with the protocols of Illumina. Raw data were filtered, corrected, and mapped to locust genome sequence via the HISAT software. Gene expression levels were measured using the criteria of reads per kb per million mapped reads. DEGs were detected using the DESeq software^66^. The *P* values were adjusted using the Benjamini and Hochberg’s approach for controlling the false discovery rate. Genes with an adjusted *P*-value <0.05 were assigned as differentially expressed. GO enrichment analysis was performed by the GOseq R package and gene length bias was corrected. KEGG enrichment was performed by the KOBAS software. Significance analysis was performed by Fisher’s Exact Test.

### RNA extraction and qPCR

Total RNA was extracted from the thoracic muscle or brain by using TRIzol reagent (Invitrogen, USA). The relative expression of mRNA was quantified with SYBR Green 1 Master Mix (Roche, USA) and LightCycler 480 instrument (Roche, USA). Five biological replicates were assayed for statistical analysis. *Rp49* was considered as endogenous controls for mRNAs. qPCR primers are listed in Supplementary Table 8.

### Western blot

Total proteins of the thoracic muscle were extracted using TRIzol reagent. The proteins were subjected to 10% polyacrylamide gel electrophoresis and transferred to polyvinylidene difluoride membranes (Millipore, USA). The membranes were then blocked in 3–5% (wt/vol) skimmed milk at room temperature (RT) for 2 h, followed by incubation with primary antibody (anti-PTP1B, 1:2000; anti-tubulin, 1:5000) in 3% (wt/vol) skimmed milk at RT for 2 h or at 4 °C overnight and with secondary antibody (1:5000; ComWin, CW0234S, China) at RT for 1 h. The immunological blot was detected by an ECL Western Blot Kit (Thermo Fisher Scientific, USA). All western blot assay were independently repeated for at least three times except for the RNA efficiency test (see Supplementary Fig. 15).

### Phosphorylation-level detection

The phosphorylation levels of InR and AKT were separately detected using the phosphorylation antibodies of InR (1:2000; Abcam, ab62321, USA) and AKT (1:1000; CST, 4054 S, USA) by western blot.

### Trehalose and glucose quantification

To derivatize hemolymph sample, we used 10 μl of cell-free hemolymph from three locusts previously starved for 2 h. The hemolymph was diluted with 90 μl distilled water (dH_2_O) (containing 0.1 mg mL^−1^ sucrose as an internal standard) and added to 300 μl methanol. The mixture was incubated on ice for 30 min and centrifuged at 10,000 rpm at 4 °C for 15 min. A total of 200 μl supernatant was transferred to a new EP tube and evaporated with a vacuum concentrator. The dry supernatant was then dissolved in 50 μl freshly prepared methoxylamine hydrochloride (15 mg mL^−1^ in pyridine; Sigma, USA) and incubated at RT overnight. The sample was centrifuged at 10,000 rpm for 5 min to remove the undissolved substance. Last, the supernatant was trimethylsilylated by 50 μl *N*-methyl-*N*-(trimethylsilyl)trifluoroacetamide (containing 1% trimethylchlorosilane; Sigma, USA)^67^.

The thoracic muscle from the three locusts was homogenized with 200 μl phosphate-buffered saline (PBS; containing 0.1 mg mL^−1^ sucrose) and centrifuged at 10,000 rpm at 4 °C for 15 min. A total of 180 μl supernatant was added into 240 μl methanol and incubated on ice for 30 min. The sample was centrifuged at 10,000 rpm at 4°C for 15 min. A 300 μl aliquot of the supernatant was added into 300 μl chloroform and mixed thoroughly. The sample was then centrifuged at 10,000 rpm at 4 °C for 15 min. A portion of 200 μl of the supernatant was transferred to a new EP tube and evaporated^68^. The sample was derivatized as mentioned above. Trehalose and glucose were quantified using Agilent 6890N-5973N, following the gas chromatography–mass spectrometry analysis program with slight modifications. In detail, initial temperature was held at 75 °C for 1 min, followed by 5 °C min^−1^ ramp to 250 °C for 5 min, and 5 °C min^−1^ ramp to 320 °C for 3 min. A total of 1 μl of sample was injected in split-less mode at 250 °C under helium carrier gas flow set at 1 ml min^−1^. The measured values were normalized to lysate protein levels for the tissue sample.

### Glycogen measurement

Glycogen was measured using the Glycogen Colorimetric/Fluorometric Assay Kit (Bio vison K646–100, USA) following the manufacturer’s protocols. In brief, thorax muscle from the three locusts was homogenized with 300 µl of dH_2_O and boiled for 10 min to inactivate enzymes. The samples were centrifuged at 14,000 × *g* for 15 min. The supernatant was diluted 10 times with dH_2_O for glycogen assay. The measured values were normalized against lysate protein levels.

### Acetyl-CoA measurement

Fresh thorax muscle was deproteinized by 1.0 M perchloric acid precipitation before thorough homogenization. The samples were centrifuged at 10,000 × *g* for 10 min to remove insoluble material. The supernatant was then neutralized with 3 M potassium bicarbonate solution and cooled on ice for 5 min. We ensured that pH of the solution was within the range of 6–8. Acetyl-CoA levels were measured using an Acetyl-Coenzyme A Assay Kit (Sigma, MAK039-1KT, USA) following the manufacturer’s protocol and then normalized to protein levels.

### NADH measurement

Thorax muscle was freshly homogenized with 400 µl of NADH/NAD Extraction Buffer and centrifuged for 10 min at 4 °C at 10,000 × g. The supernatant was then added to the 10-kD spin column (Abcam, ab93349, USA) and centrifuged at 10,000 × *g* for 20 min at 4 °C. The filtrate was collected and used for NADH assay via the NAD/NADH Assay Kit (Abcam, ab65348, USA). The measured values were normalized to lysate protein levels.

### Lactate measurement in hemolymph

Hemolymph samples from at least three locusts were centrifuged for 10 min at 4 °C at 1000 × g to remove the hemocyte. The supernatant was used for lactate assay using the Lactate Colorimetric/Fluorometric Assay Kit (Bio vision, K607, USA).

### RNAi

RNAi assay was performed to knock down the *PTPN1* gene expression. The dsRNA of *GFP* and *PTPN1* was prepared using T7 RiboMAX Express RNAi system (Promega, USA) following the manufacturer’s protocol. The 3 µg µl^−1^ dsRNA (9 µg) was injected into 5-day-old male adult locusts at the second ventral segment of the abdomen. A second injection of the same dose was performed after 3 days. The injected locusts were then placed into the hypoxic chamber for hypoxia treatment. After 5 days, *PTPN1* expression levels were examined by qPCR and western blot. The primers for *PTPN1* dsRNA synthesis are listed in Supplementary Table 8.

### Recombinant expression and purification

Wild- and mutant-type locust PTP1B were recombinantly expressed in *Escherichia coli*. The coding sequences of the above two proteins were amplified from locust muscle tissue using the primers PTP1BexF1 and R1. The PCR products were then cloned into pET32a (Novagen, Germany) vector. The recombinant proteins were purified by affinity chromatography using His-Bind resin (GE Healthcare, USA) following the manufacturer’s instructions. The primers for *PTPN1* cDNA amplification are listed in Supplementary Table 8.

### PTP1B enzyme activity assay

PTP1B enzyme activity was measured using the PTP Assay Kit 2 (Millipore, 17–126, USA) following the manufacturer’s protocols. For the in vivo assay, the thoracic muscle of locusts was thoroughly homogenized and centrifuged at 10,000 × *g* for 20 min to remove the insoluble material. The samples were then added to a 10-kD spin column (Abcam, ab93349, USA) and centrifuged at 14,000 × *g* for 15 min at 4 °C to eliminate native phosphate. After centrifugation, the samples were re-suspended with 100 µl phosphatase extraction buffer. A total of 5 µl supernatant was used for the enzyme activity assay. The measured values were normalized to lysate protein levels. For the in vitro assay, mutated and wild-type PTP1B were separately overexpressed in *Drosophila* S2 cells (ATCC CRL-1963). After 36 h, the transfected cells were treated with 1% hypoxia for 6 h. PTP1B enzyme activity was then measured by PTP Assay Kit according to the protocols. The measured values were normalized to lysate protein levels. For recombinant protein, 0.5 µg purified recombinant PTP1B and BSA were used for enzyme activity measurement.

### TUNEL (terminal deoxinucleotidyl transferase-mediated dUTP-fluorescein nick end labeling) staining assay

Tissue sections were obtained from the thoracic muscle of 5-day-old male locusts. The locusts were first subjected to severe hypoxia (2% *P*o_2_) for 6 h. In the trehalose rescue group, locusts were injected with 500 µg of trehalose solution (100 µg µl^−1^ in ddH_2_O) before hypoxic treatment. TUNEL staining was performed with the In Situ Cell Death Detection Kit (Roche, 11684817910, USA) following the manufacturer’s instructions. For details, thoracic muscle tissue sections were fixed with 4% paraformaldehyde for 20 min and washed with PBS for 30 min. The fixed tissue sections were then permeabilized with 0.1% TritonX-100 for 10 min at RT and treated with 3 U ml^−1^ DNase1 for 10 min to break the DNA strands. The tissue sections were then sequentially incubated with a TNNEL reaction mixture and 0.5% Hoechst (Life, H3570, USA) before washing with PBS three times. The tissue sections were imaged using an LSM 710 confocal fluorescence microscope (Zeiss) at a ×10 magnification. The experiments were repeated at least five times. TUNEL intensity was measured and quantified using Image J.

### Lifespan assay

Five days after molting, a total of 120 male locust adults were selected for lifespan measurement. The locusts were kept in four cages with 30 individuals each. Two of the cages were placed under normoxic condition. The other two cages were placed in a hypoxic chamber at *P*o_2_ = 10 kPa. The locusts were injected with dsRNA three times at a 7-day interval. Survival curves were analyzed using Wilcoxon rank-sum test.

## Electronic supplementary material

Supplementary information

Reporting Summary

## Data Availability

The published reference genome of migratory locust used for mapping is available at LocustBase [http://159.226.67.243/download.htm]. Fastq files of the genome sequence for each of 24 locusts are available at BioProject PRJNA433455. Fastq files of the transcriptome sequence for hypoxia treatment are available at BioProject PRJNA438378. Fastq files of the transcriptome sequence for gene expression in different tissues are available at BioProject PRJNA436219. The GenBank accession number for the mRNA sequence of locust PTP1B is MH973608. A reporting summary for this article is available as a Supplementary Information file.
