# Supplementary material for: Genetic variation in PTPN1 contributes to metabolic adaptation to high-altitude hypoxia in Tibetan migratory locusts
Source: Nat Commun. 2018 Nov 26;9:4991. doi: 10.1038/s41467-018-07529-8 (PMC6255802; doi:10.1038/s41467-018-07529-8)
Supplement: Supplementary file 1 — Supplementary information [file 41467_2018_7529_MOESM1_ESM.pdf]

Supplementary information

Genetic variation in *PTPN1* contributes to Metabolic Adaptation to High-Altitude Hypoxia in Tibetan Migratory Locusts

Ding et al.

Supplementary Table 1. Locust sample information.

Sample ID	Locality	Altitudes	Sampling date (year)
**Tibetan-1**	Maizhikungga	4,000 m	2005
**Tibetan-2**	Maizhikungga	4,000 m	2005
**Tibetan-3**	Maizhikungga	4,000 m	2005
**Tibetan-4**	Lhasa	3,700 m	2014
**Tibetan-5**	Lhasa	3,700 m	2014
**Tibetan-6**	Lhasa	3,700 m	2014
**Tibetan-7**	Lhasa	3,700 m	2014
**Tibetan-8**	Lhasa	3,700 m	2014
**Tibetan-9**	Lhasa	3,700 m	2014
**Tibetan-10**	Shannan Nedong	4,000 m	2015
**Tibetan-11**	Doilung Deqing	3,700 m	2005
**Tibetan-12**	Doilung Deqing	4,000 m	2015
**HaiN-1**	Dongfang	≤ 20 m	2014
**HaiN-2**	Dongfang	≤ 20 m	2014
**HaiN-4**	Dongfang	≤ 20 m	2014
**HaiN-5**	Dongfang	≤ 20 m	2014
**HaiN-6**	Danzhou	≤ 20 m	2014
**HaiN-7**	Danzhou	≤20 m	2014
**HaiN-9**	Danzhou	≤ 20 m	2014
**HaiN-11**	Danzhou	≤ 20 m	2014
**HaiN-19**	Danzhou	≤ 20 m	2014
**HaiN-21**	Danzhou	≤ 20 m	2014
**TJ**	Dagang	≤ 20 m	2014
**LN**	Huludao	≤ 20 m	2014

Supplementary Table 2. Summary of whole-genome sequencing data

Samples	Mismatch rate	Mapping rate	Average depth	Coverage 1X	Coverage 4X	Coverage 10X
**Tibetan-1**	2.70%	96.14%	9.74	87.01%	71.97%	28.98%
**Tibetan-2**	2.61%	96.55%	12.66	87.99%	78.08%	45.37%
**Tibetan-3**	2.94%	95.49%	12.31	88.37%	78.01%	45.05%
**Tibetan-4**	2.71%	92.79%	10.59	87.31%	73.09%	33.83%
**Tibetan-5**	2.73%	96.27%	10.7	87.03%	73.52%	35.73%
**Tibetan-6**	2.61%	96.18%	10.84	86.98%	73.00%	34.74%
**Tibetan-7**	2.64%	95.43%	10.77	87.46%	73.56%	36.19%
**Tibetan-8**	2.71%	96.17%	10.97	87.67%	74.87%	37.29%
**Tibetan-9**	2.66%	86.12%	10.94	87.67%	75.09%	36.81%
**Tibetan-10**	2.73%	96.09%	10.79	87.49%	75.07%	37.10%
**Tibetan-11**	2.63%	93.31%	9.86	87.07%	72.01%	28.59%
**Tibetan-12**	2.57%	96.34%	10.92	87.53%	75.01%	37.20%
**HaiN-1**	2.78%	64.17%	10.73	87.91%	74.75%	35.68%
**HaiN-2**	2.94%	76.71%	10.71	87.65%	74.45%	36.47%
**HaiN-4**	2.78%	91.11%	10.54	87.88%	74.45%	35.11%
**HaiN-5**	2.81%	81.81%	10.69	87.90%	74.83%	35.64%
**HaiN-6**	2.75%	89.40%	10.52	88.04%	75.70%	35.61%
**HaiN-7**	2.72%	76.65%	10.48	87.83%	75.12%	33.38%
**HaiN-9**	2.90%	93.44%	10.89	87.94%	75.89%	37.14%
**HaiN-11**	2.76%	71.99%	10.04	87.69%	74.04%	31.33%
**HaiN-19**	3.06%	95.04%	12.02	88.10%	77.76%	43.92%
**HaiN-21**	2.76%	96.32%	10.95	87.95%	75.23%	36.46%
**TJ**	2.44%	96.79%	13.22	89.75%	79.15%	46.68%
**LN**	2.39%	95.93%	10.87	89.42%	78.08%	38.41%

Supplementary Table 3. Gene Ontology (GO) enrichment of positively selected genes

ID	Term Type	Description	*n*	*P* Value
**GO:0051186**	BP	Cofactor metabolic process	6	0.014846
**GO:0048869**	BP	Cellular developmental process	3	0.018246
**GO:0051188**	BP	Cofactor biosynthetic process	5	0.020146
**GO:0048856**	BP	Anatomical structure development	3	0.026556
**GO:0009889**	BP	Regulation of biosynthetic process	18	0.028479
**GO:0006733**	BP	Oxidoreduction coenzyme metabolic process	3	0.029702
**GO:0008104**	BP	Protein localization	14	0.030984
**GO:0006732**	BP	Coenzyme metabolic process	5	0.032354
**GO:0009058**	BP	Biosynthetic process	49	0.035345
**GO:0044767**	BP	Single-organism developmental process	3	0.03784

Note: Only GO terms with more than 2 genes are shown, *n* represents the number of genes and BP represents biological process.

Supplementary Table 4. Top 20 genes with the highest ZF_{ST} value

Gene ID	Name	Description	**Z*F*$_{ST}$**
**LOCMI16039**	*sdhd-1*	Putative succinate dehydrogenase [ubiquinone] cytochrome b small subunit, mitochondrial	5.48
**LOCMI09989**	*USP4*	Ubiquitin carboxyl-terminal hydrolase 4	5.16
**LOCMI16005**	*Fasn2*	Fatty acid synthase	5.16
**LOCMI16361**	*Ap3d1*	AP-3 complex subunit delta-1	5.03
**LOCMI07679**	*VKR*	Venus kinase receptor	4.92
**LOCMI15451**	*Osa*	Trithorax group protein osa	4.86
**LOCMI07281**	*Pof*	Protein painting of fourth	4.84
**LOCMI13746**	*If*	Integrin alpha-PS2	4.83
**LOCMI09680**	*Gas2l1*	GAS2-like protein 1	4.83
**LOCMI06683**	*rbm26*	RNA-binding protein 26	4.78
**LOCMI16009**	*Ndufb7*	NADH dehydrogenase [ubiquinone] 1 beta subcomplex subunit 7	4.75
**LOCMI14495**	*Cul1*	Cullin-1	4.72
**LOCMI16386**	*Su(dx)*	E3 ubiquitin-protein ligase Su(dx)	4.70
**LOCMI07987**	*Klhdc1*	Kelch domain-containing protein 1	4.69
**LOCMI12387**	*Rbm42*	RNA-binding protein 42	4.68
**LOCMI15987**	*Fasn1*	Fatty acid synthase	4.63
**LOCMI12038**	*COL11A2*	Collagen alpha-2(XI) chain	4.58
**LOCMI04142**	*Gphn*	Gephyrin	4.58
**LOCMI13542**	*ZC3H12C*	Probable ribonuclease ZC3H12C	4.56
**LOCMI05909**	*FLNA*	Filamin-A	4.68

Supplementary Table 5. Top 20 genes with the highest XP-CLR score

Gene ID	Name	Description	XPCLR
**LOCMI07893**	*stau2*	Double-stranded RNA-binding protein Staufen homolog 2	129.77
**LOCMI13788**	*CAPRIN1*	Caprin-1	124.27
**LOCMI08902**	*MDN1*	Midasin	121.69
**LOCMI05473**	*CAP1*	Adenylyl cyclase-associated protein 1	118.16
**LOCMI11968**	*tamm41*	Mitochondrial translocator assembly and maintenance protein 41 homolog	113.18
**LOCMI10229**	*ABCG1*	ATP-binding cassette sub-family G member 1	111.66
**LOCMI11591**	*vac14*	VAC14-like protein	98.48
**LOCMI07509**	*naprt*	Nicotinate phosphoribosyltransferase	98.06
**LOCMI04670**	*D1Pas1*	Putative ATP-dependent RNA helicase Pl10	96.64
**LOCMI10276**	*WDR74*	WD repeat-containing protein 74	90.28
**LOCMI05539**	*PTPN1*	Protein-tyrosine phosphatase-1B	89.60
**LOCMI10185**	*HERC1*	Probable E3 ubiquitin-protein ligase HERC1	88.82
**LOCMI17208**	*proPo-A3*	Phenoloxidase subunit A3	88.35
**LOCMI09847**	*Zdhhc8*	Probable palmitoyltransferase ZDHHC8	87.93
**LOCMI15739**	*GRIK3*	Glutamate receptor ionotropic, kainate 3	87.84
**LOCMI13580**	*Ctdspl2*	CTD small phosphatase-like protein 2	87.67
**LOCMI15789**	*Taldo1*	Transaldolase	86.25
**LOCMI14383**	*Efhc1*	EF-hand domain-containing protein 1	85.66
**LOCMI13361**	*SLC18B1*	MFS-type transporter SLC18B1	84.76
**LOCMI10336**	*Stathmin*	Stathmin	82.87

Supplementary Table 6. Top 20 genes with the highest ΔZHp (ZHp $_{Lowland}$ – ZHp $_{Tibetan}$) value

Gene ID	Name	Description	ΔZHp
**LOCMI14887**	*Znf235*	Zinc finger protein 235	8.59
**LOCMI14890**	*FAM103A1*	RNMT-activating mini protein	8.12
**LOCMI07893**	*stau2*	Double-stranded RNA-binding protein Staufen homolog 2	7.76
**LOCMI09930**	*DNAH3*	Dynein heavy chain 3, axonemal	7.69
**LOCMI06546**	*Osbpl1a*	Oxysterol-binding protein-related protein 1	7.63
**LOCMI06820**	*DNAH3*	Dynein heavy chain 3, axonemal	7.45
**LOCMI09748**	*CLCC1*	Chloride channel CLIC-like protein 1	7.14
**LOCMI05473**	*CAP1*	Adenylyl cyclase-associated protein 1	7.09
**LOCMI06460**	*Xpnpep3*	Probable Xaa-Pro aminopeptidase 3	7.00
**LOCMI11179**	*RPUSD1*	RNA pseudouridylate synthase domain-containing protein 1	6.95
**LOCMI14781**	*Rnf157*	RING finger protein 157	6.94
**LOCMI08902**	*MDN1*	Midasin	6.85
**LOCMI13561**	*CHD1*	Chromodomain-helicase-DNA-binding protein 1	6.81
**LOCMI17242**	*Adck1*	Uncharacterized aarF domain-containing protein kinase 1	6.76
**LOCMI07155**	*Aggf1*	Angiogenic factor with G patch and FHA domains 1	6.69
**LOCMI09680**	*Gas2l1*	GAS2-like protein 1	6.66
**LOCMI13361**	*SLC18B1*	MFS-type transporter SLC18B1	6.66
**LOCMI13651**	*FAM120B*	Constitutive coactivator of peroxisome proliferator-activated receptor gamma	6.60
**LOCMI06948**	*Rpp40*	Ribonuclease P, Rpp40	6.58
**LOCMI04195**	*ilvbl*	Acetolactate synthase-like protein	6.49

Supplementary Table 7. Nonsynonymous mutations of PSGs involved in energy metabolism

Gene	Nonsynonymous mutations
PTPN1	p.Asn349Ile (c.1046A>T)
FAS1	p.Mel1242Leu (c.3724A>T)
	p.Ala675Gly (c.2024C>G)
	p.Lys664Arg (c.1991A>G)
FAS2	non
FAS3	p.Ser508Gly (c.1522A>G)
	p.Ala537Pro (c.1609G>C)
	p.Gln699Arg (c.2096A>G)
Ndufb7	non
PIK3CD	non
ADIPOR1	non
FAR1	non
PPT1	p.Ile13Met (c.39A>G)
ACO1	non
SDHD1	non
ATP1A2	p.Ser137Ala (c.409T>G)
VLCAD	non

Note: Only nonsynonymous mutations with $F_{ST} > 0.5$ are shown.

Supplementary Table 8. Primers used in this study

Primer name	Sequence, 5'-3'	Description
PTP1BF	ATGAGCAGGACTCCTGGTAA	Clone the full length of PTP1B
PTP1BR	TCAAACACGGTTAGAGAAGTA	
PTP1BT7F	TAATACGACTCACTATAGGTATACTACATGGCCTGACTT	*PTPN1* dsRNA
PTP1BT7R	TAATACGACTCACTATAGGTCTGCAGAGACCTCAGGTTC	
PTP1BexF1	TATCGAATTCATGAGCAGGACTCCTGGTAA	Overexpression
PTP1BexR1	TATCAAGCTTTCAAACACGGTTAGAGAAGTA	
PTP1BexF2	TATCGAATTCGCCATGGGCAGGACTCCTGGTAA	
PTP1BexR2	TATCGCGGCCGCCAACACGGTTAGAGAAGTAAC	
PTP1BrtF	AGCCAACAAACCACTCCCTG	qPCR
PTP1BrtR	TCATCATCACCTGCTTCCTTA	
IlprtF	ATGGACTGACCAGCCCCTAA	
IlprtR	CGGCTCCATCTCCTAAAACAAA	
ACA rtF	TGATTTATCTGTCGGCTCCTA	
ACA rtR	GAGACCTGCAATATGGTATGA	
ACC rtF	CATTGCCGAGCATTCATCGTG	
ACC rtR	GGCCGTAATTGAGTCAACAGG	
PDH E3rtF	TGGAATGTGATGTCCTGCTCG	
PDH E3rtR	CCTCATCTTCAGCTTTGTGGG	
ETIF rtF	ACCATATCAAAGCAGCAAGCG	
ETIF rtR	TTAATCAACCAACGTCCACCC	
GYS rtF	TGCATTCCTGATATTCCCTACC	
GYS rtR	AATCTCATACATCCGCTTTCC	
PDHE1brtF	GATTCTCCAGTTATCCGTGTCA	
PDHE1brtR	TCGCTCTGCTCACTTCCTCA	
PYKrtF	CAGGCATGAATGTAGCGAGGTT	
PYKrtR	CCCTTCAAGTAGCCCTGTTCT	
RPS6rtF	AGGCGAGATGGTGAAAGAAAG	
RPS6rtR	AAACGACGAGGAATCGTATTG	
Ald1rtF	ACTCAGGGACTCGACGACTTG	
Ald1rtR	GCGTGCCAGAACATTAGCATT	
Ald2rtF	TGCTGGGATTGCTTTCCTTTC	
Ald2rtR	CTCGCTTCTTAAACTCCTCTTGG	
HexrtF	GGCCTTAATCATGCAACAGAC	
HexrtR	AGCCATCAATGCCCACTGTAA	
PTIrtF	GGCGGGCCTTTCGATTATT	
PTIrtR	GGCTCCAGTCCGTTATCTTGT	

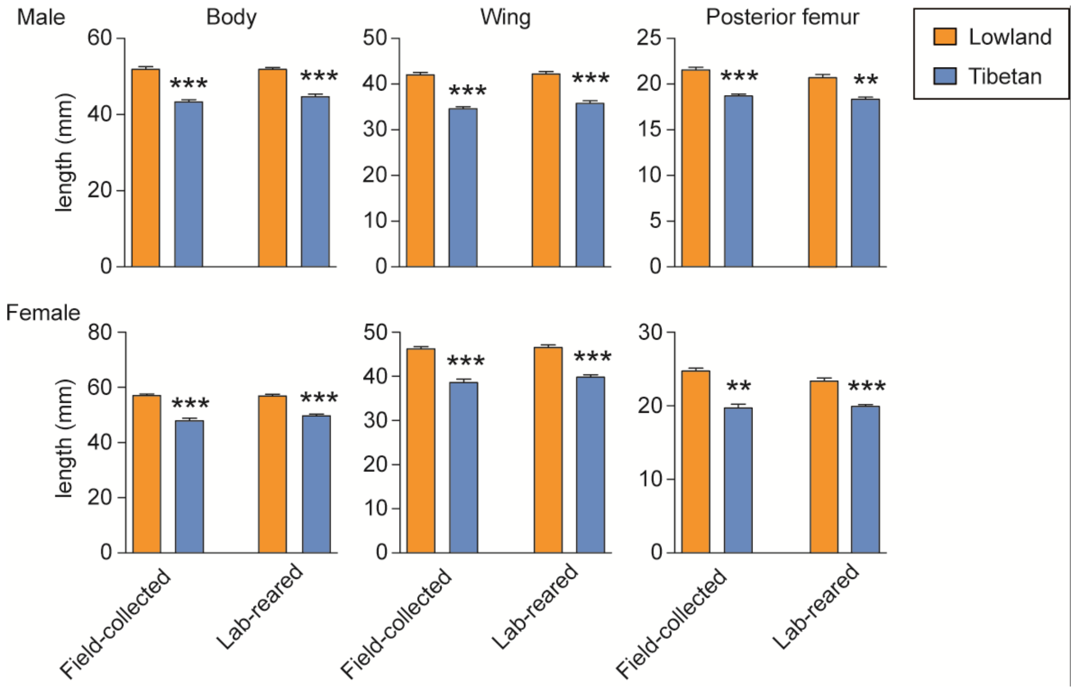

Supplementary Figure 1. Difference in body size between the Tibetan and lowland locusts. *Field-collected* represents sexually mature adults newly collected from the field. *Lab-reared* represents sexually mature adults that had been reared in laboratory under normoxic condition for two generations. Measurements are shown as mean ± standard error (s.e.m.). $^{**}P < 0.01$, $^{***}P < 0.001$ (Student's *t*-test). These samples are selected randomly from stock locust populations. Sample size: $n \geq 8$ individuals.

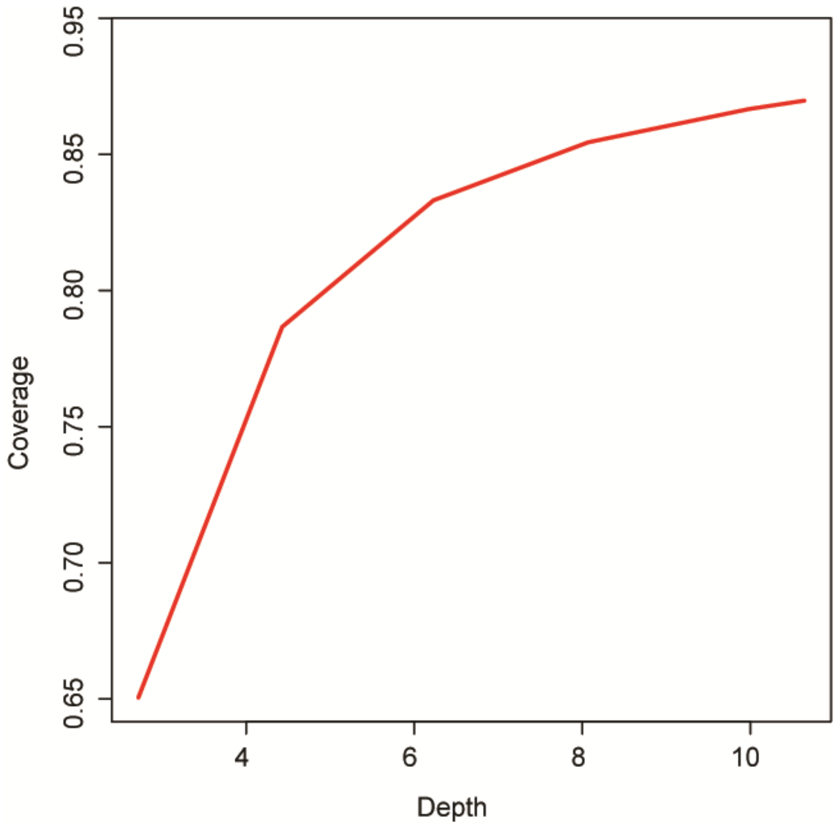

Supplementary Figure 2. Saturation curve of coverage rate and sequencing depth based on the whole-genome sequence data of a Tibetan locust.

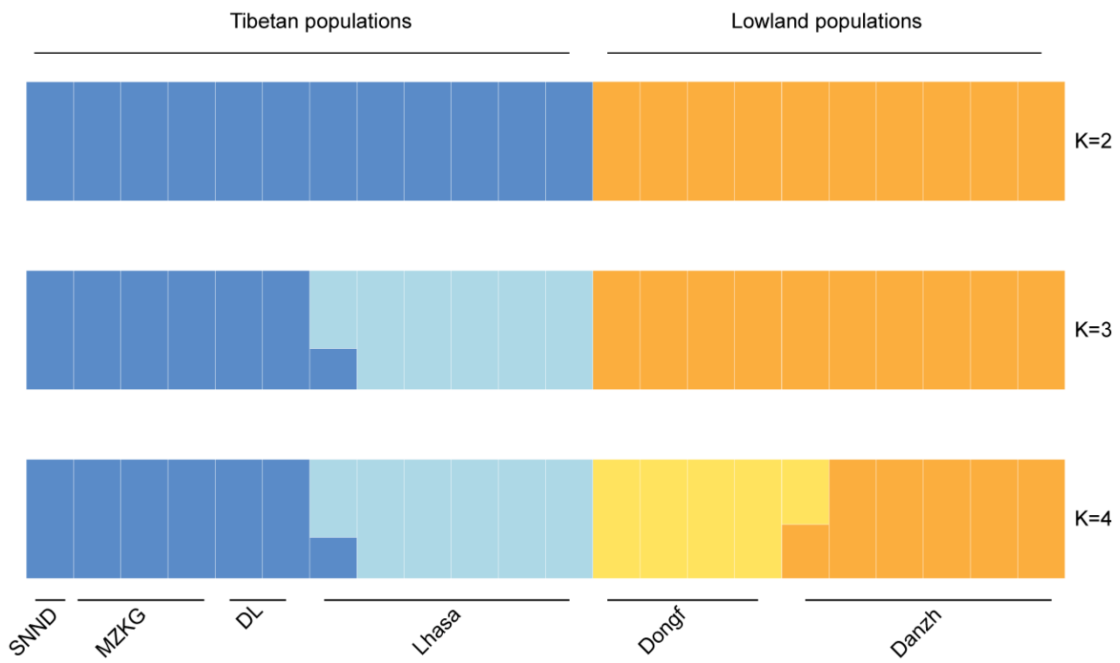

Supplementary Figure 3. Genetic structure analysis of Tibetan and lowland locusts from K = 2–4.

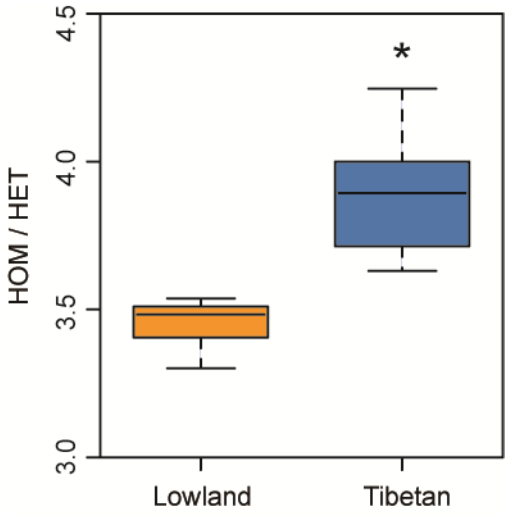

Supplementary Figure 4. Heterozygosity across populations. Shown is estimated ratio of homozygous SNPs (HOM) to heterozygous SNPs (HET) /individual. * $P < 0.05$ (Student's *t*-test). The center lie of boxplots represents median value, the bounds of the box represents 75th and 25th percentile and the whiskers represent maximum and minimum value.

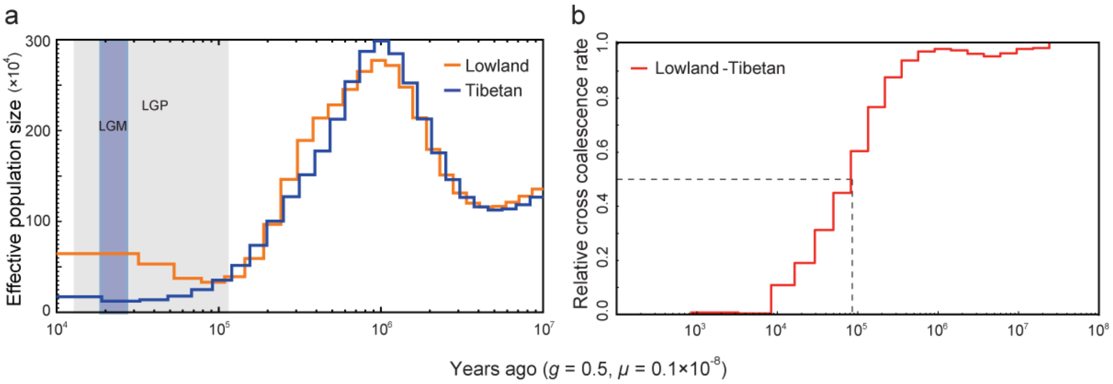

Supplementary Figure 5. Demographic histories of the locusts. (a) Effective population size of the Tibetan and lowland locust. LGM represents the last glacial maximum, LGP represents the last glacial period. (b) Relative cross coalescence rates between Tibetan and lowland locusts. g, generation time; μ, neutral mutation rate per generation.

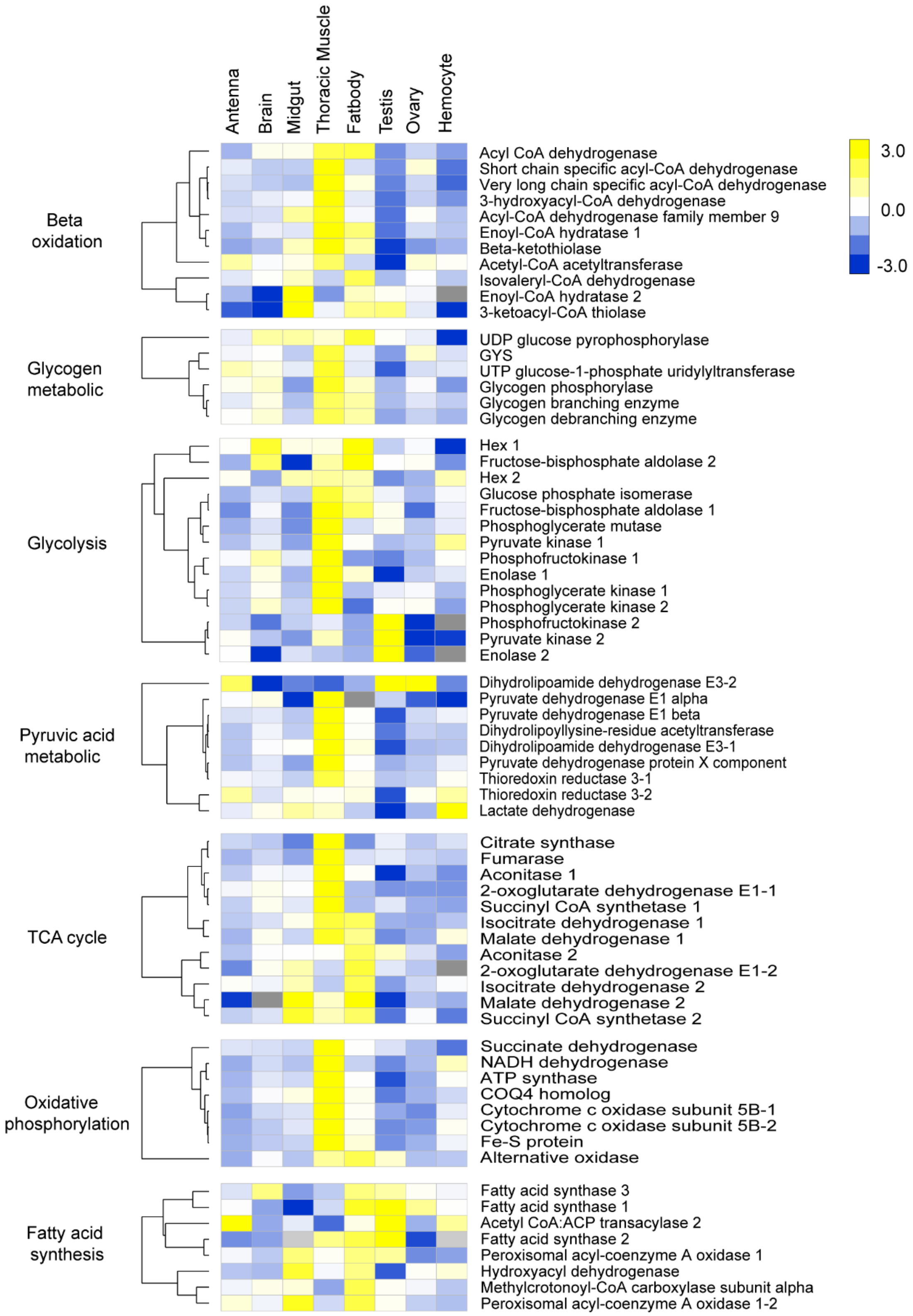

Supplementary Figure 6. Expression of metabolism-related genes in different tissues. Heat map signal indicates log2 fold-change values relative to the mean expression level within each group. Yellow signal represents higher expression and blue represents lower expression relative to the mean expression level within the group. Expression levels were measured through transcriptome sequencing.

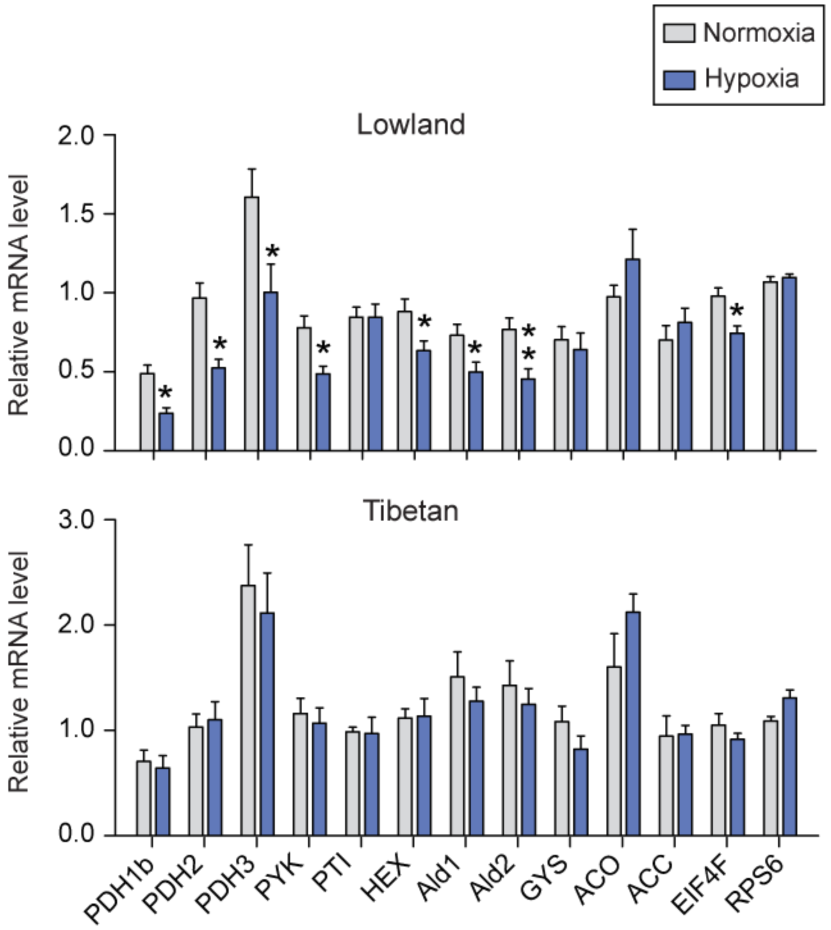

Supplementary Figure 7 Expression levels of metabolism-related genes in response to hypoxia. These genes are involved in glycolysis, pyruvic acid metabolism, fatty-acid synthesis and protein synthesis. mRNA level was measured by quantitative real-time PCR and normalized against that of the internal control *rp49*. The values are expressed in mean ± s.e.m. * $P < 0.05$, ** $P < 0.01$ (Student's *t*-test) compared to control (i.e., normoxia) ($n = 5$ replicates).

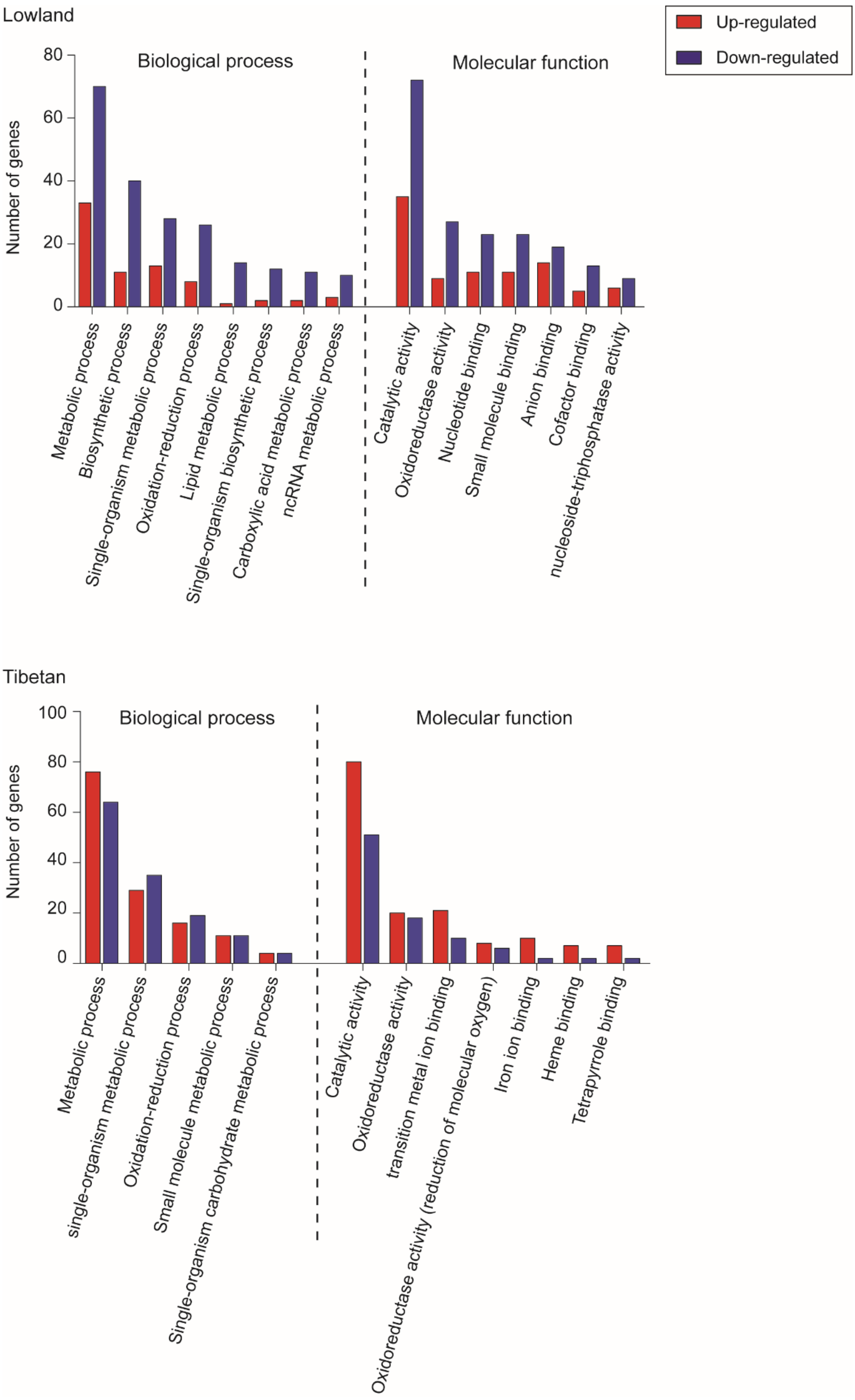

Supplementary Figure 8. GO enrichment of differentially expressed genes in response to hypoxia exposure of locust adults.

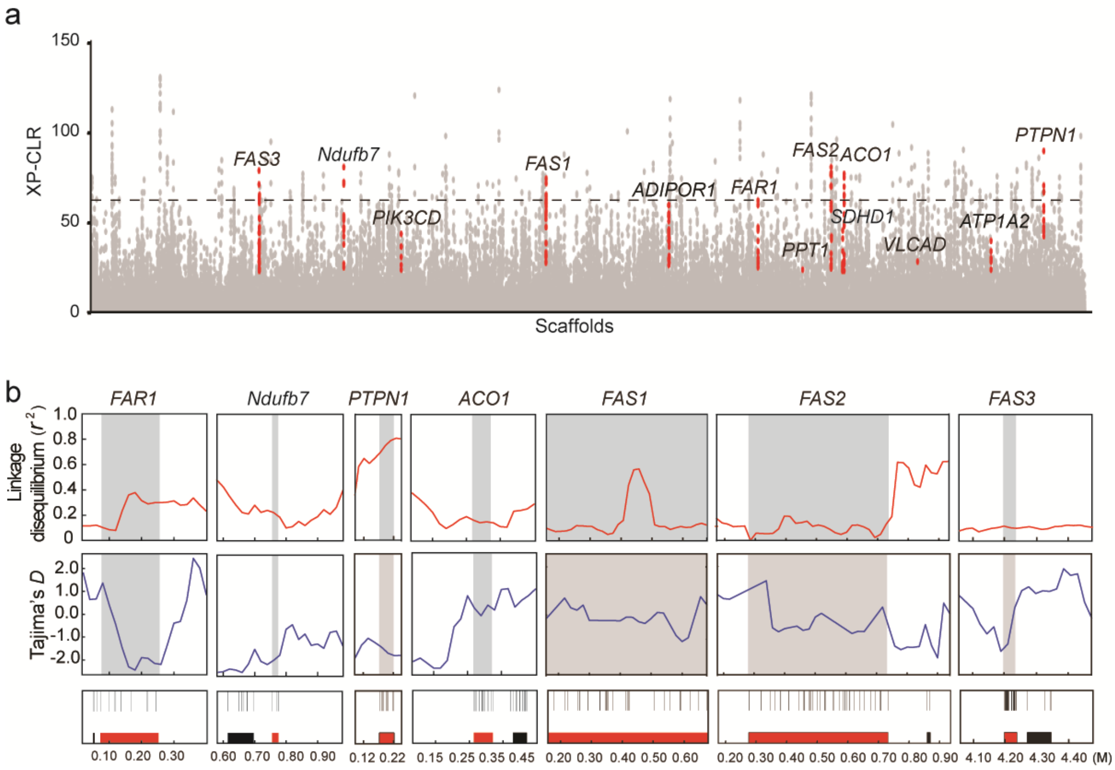

Supplementary Figure 9. Selective sweep signal of PSGs involved in energy metabolism. PSG, positively selected genes. (a) Genomic landscape of the XP-CLR values. All the scaffolds were arranged end to end in random order, as shown on the x-axis. Each point represents a 100-kb sliding window. Red colored points indicate gene regions. The region above the dashed line is top 0.5% XP-CLR statistics. (b) Tajima's *D* and average LD values of Tibetan locusts' genome. Data are plotted using a 40-kb sliding window in a scaffold. The red regions at bottom are the gene regions of the PSGs.

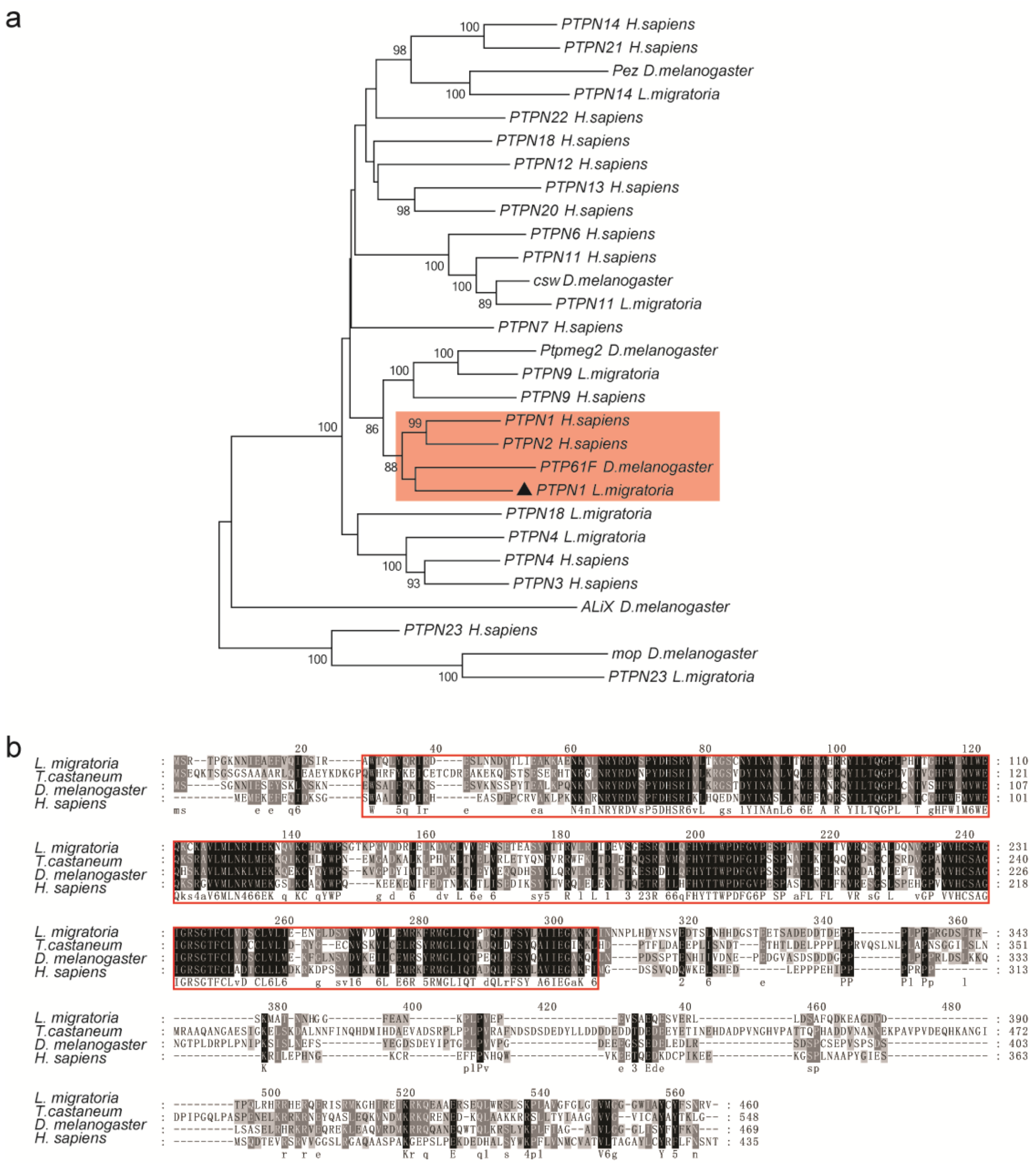

Supplementary Figure 10. Phylogenetic tree analysis and sequence alignment of PTP1B. (a) Phylogenetic tree construction of PTP1B. MEGA 5.0 was used for phylogenetic tree analysis with the corresponding amino acid sequences of non-transmembrane PTP family genes from *Homo sapiens*, *Drosophila melanogaster* and *Locusta migratoria*. One thousand bootstraps were performed. Only bootstrap values >85 are shown. (b) Amino acid alignment of the PTP1B in different species. The alignment among *L. migratoria*, *Tribolium castaneum*, *D. melanogaster* and *H. sapiens* was performed using MEGA 5.0 and GENEDOC. The region in red box represents PTP domain.

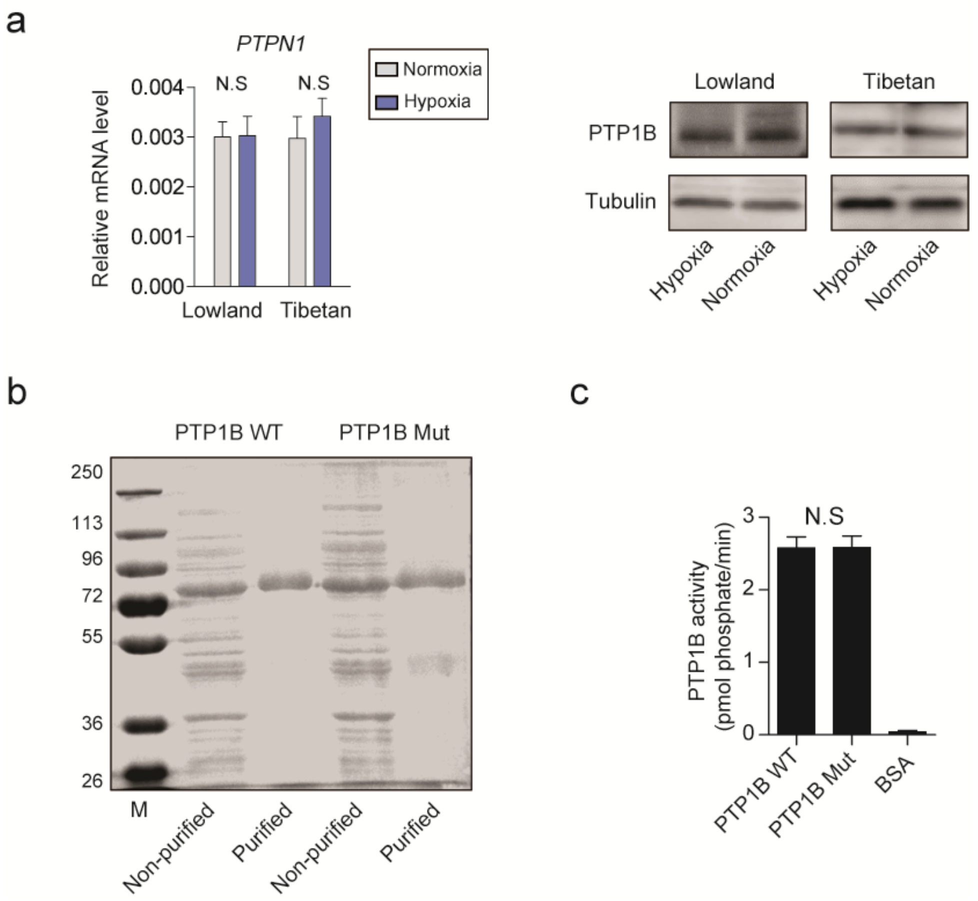

Supplementary Figure 11. Protein level detection and enzyme activity assay of locust PTP1B. (a) Quantitative real-time PCR and western blot detecting the mRNA and protein level of *PTPN1* in the Tibetan and lowland locusts in vivo. Supplementary Figs. 15a and 15b show the original images. (b) Expression and purification of locust PTP1B recombinant protein in S2 cells. (c) The enzyme activity of the recombinant locust PTP1B under normoxic condition, Bovine serum albumin (BSA) is used as negative control. WT represents wild-type PTP1B, whereas Mut represents mutant-type PTP1B. N.S represents no significant difference. The values are expressed as mean ± s.e.m (*n* = 3 replicates). Supplementary Fig. 15c shows the original image.

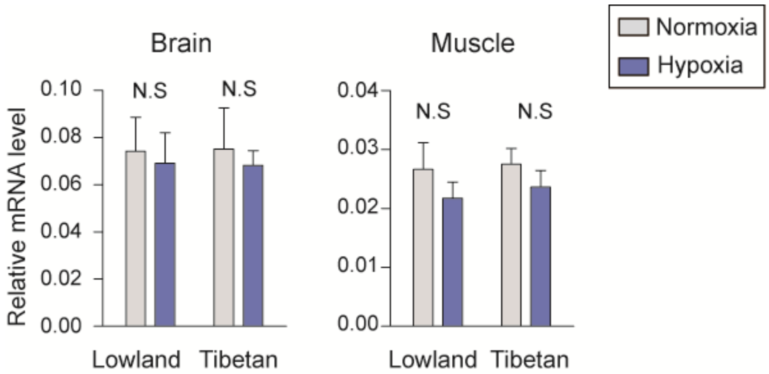

Supplementary Figure 12. mRNA levels of locust insulin like peptides in brain and thoracic muscle after hypoxia induction. The mRNA level was normalized against that of the internal control *rp49*. The values are expressed as mean ± s.e.m. N.S. represents no significant difference compared with normoxia (n = 5 replicates).

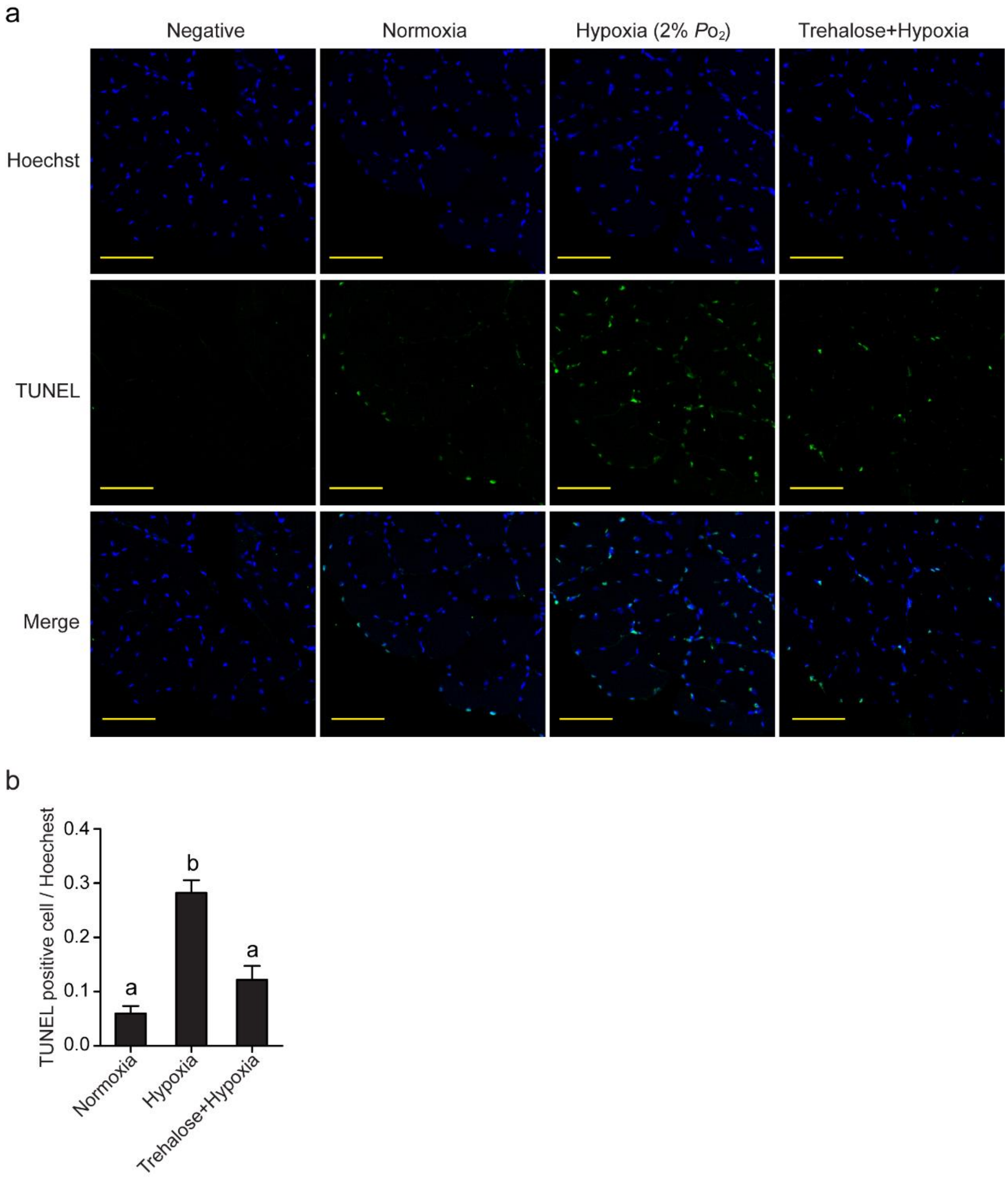

Supplementary Figure 13. In situ cell death detection assay. The cell death level of myocyte was tested by an in situ cell death detection kit. Cell nuclei were stained in blue using Hoechst. DNA fragments in dead cell nuclei were stained in green using TUNEL. Locust adult was injected with 100 μg/μl trehalose solution in trehalose treatment. Normoxia represents 21% Po_2. Hypoxia represents exposure to 2% Po_2 for 6h. Bar length = 50 μm. (b) Percentage of dead cells represented as the TUNEL/ Hoechst ratio. The values are expressed as mean ± s.e.m. Significant differences are denoted by different letters (one-way ANOVA, $P < 0.05$) ($n = 5$ replicates).

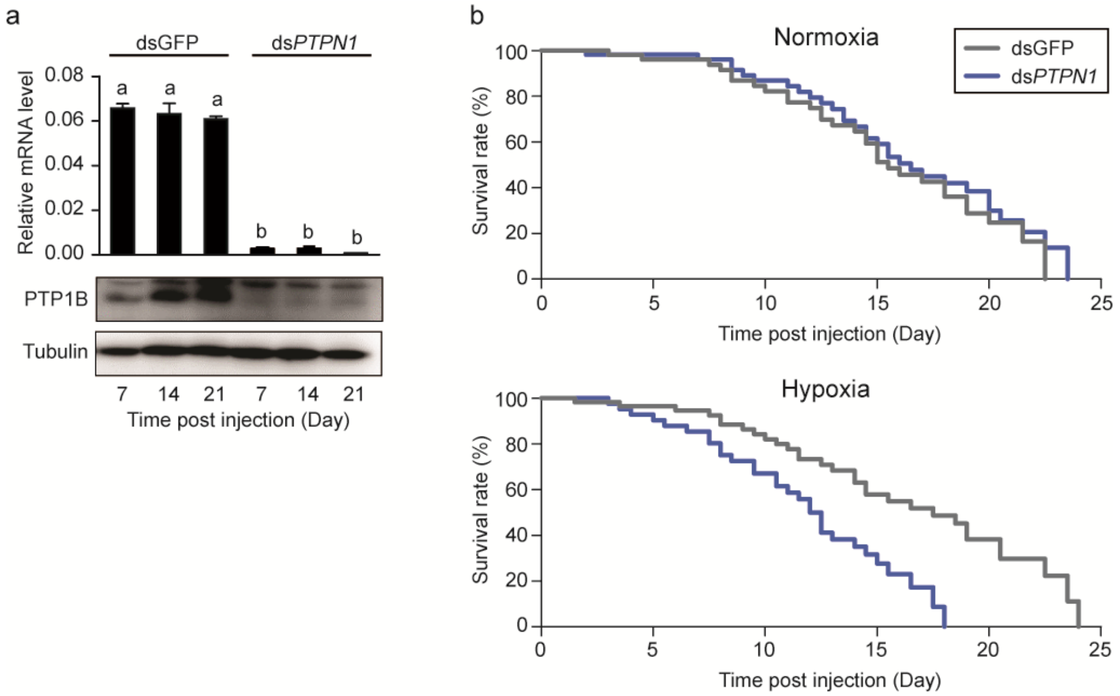

Supplementary Figure 14. Effects of *PTPN1* knockdown on adult lifespan. (a) RNAi efficiency in *PTPN1* expression knockdown. The mRNA and protein expression level of *PTPN1* in thoracic muscle was measured by quantitative real-time PCR and western blot, respectively. Double strand RNA (dsRNA) was injected to knock down expression. ds*GFP* was used as control. The values are expressed as mean ± s.e.m. Significant differences are denoted by different letters (one-way ANOVA, $P < 0.05$). Supplementary Fig. 15l shows the original image. (b) Effect of *PTPN1* knock-down on survivorship curves of locust adult under normoxia and hypoxia condition. Survival was recorded from 5-d-old adults when first dsRNA was injected. Wilcoxon rank sum test was used to test the significance of difference. $n = 30$. All assays were repeated twice with similar results.

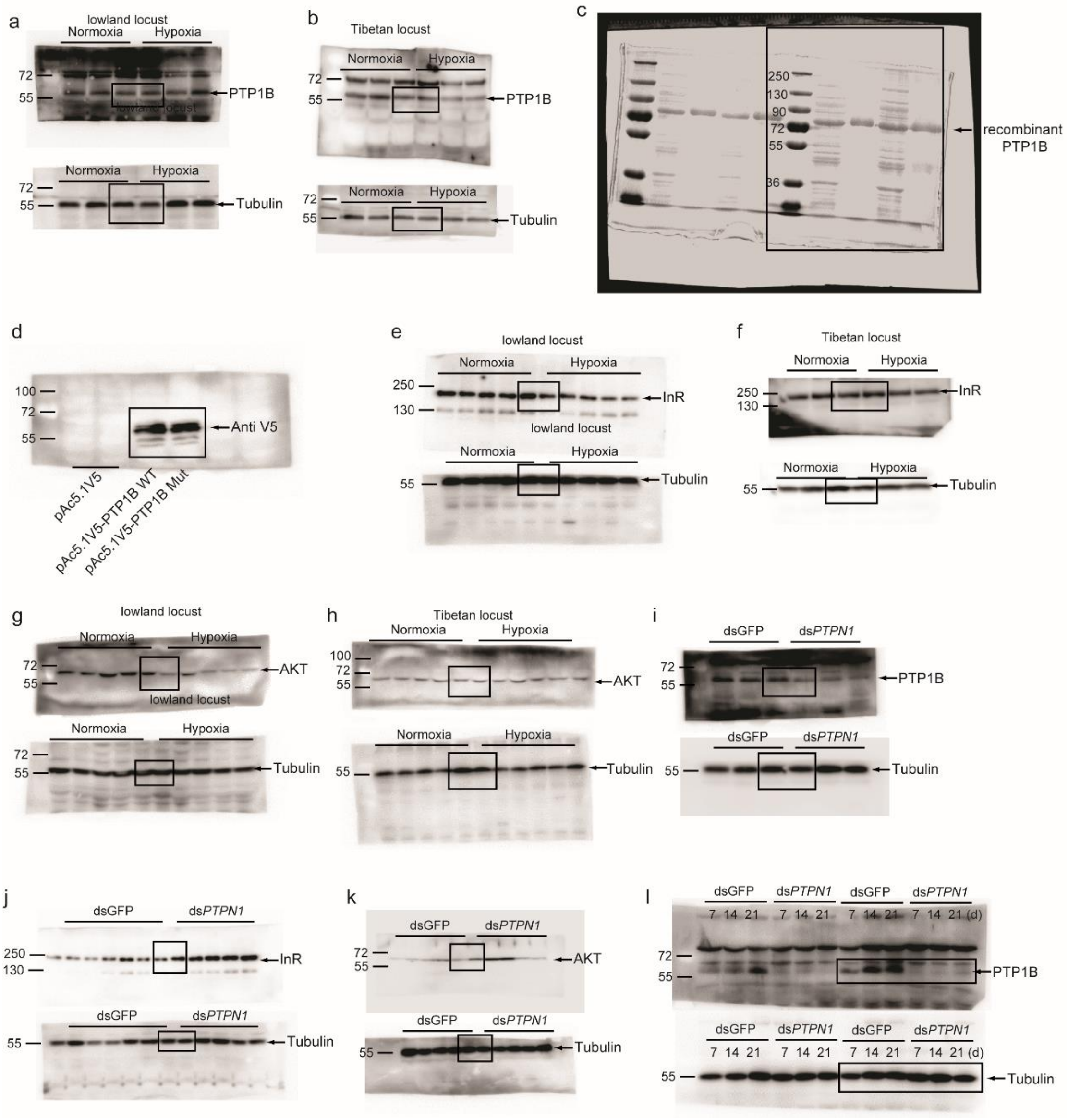

Supplementary Figure 15. The full-size western blot and SDS-PAGE scan.
