# Supplementary material for: Genetic variation in PTPN1 contributes to metabolic adaptation to high-altitude hypoxia in Tibetan migratory locusts
Source: Nat Commun. 2018 Nov 26;9:4991. doi: 10.1038/s41467-018-07529-8 (PMC6255802; doi:10.1038/s41467-018-07529-8)
Supplement: Supplementary file 2 — Reporting Summary [file 41467_2018_7529_MOESM2_ESM.pdf]

nature research

Corresponding author(s): Bing Chen and Le Kang

Reporting Summary

Nature Research wishes to improve the reproducibility of the work that we publish. This form provides structure for consistency and transparency in reporting. For further information on Nature Research policies, see Authors & Referees and the Editorial Policy Checklist.

Statistical parameters

When statistical analyses are reported, confirm that the following items are present in the relevant location (e.g. figure legend, table legend, main text, or Methods section).

n/a	Confirmed	
☐	☒	The exact sample size (*n*) for each experimental group/condition, given as a discrete number and unit of measurement
☐	☒	An indication of whether measurements were taken from distinct samples or whether the same sample was measured repeatedly
☐	☒	The statistical test(s) used AND whether they are one- or two-sided<br>*Only common tests should be described solely by name; describe more complex techniques in the Methods section.*
☐	☒	A description of all covariates tested
☐	☒	A description of any assumptions or corrections, such as tests of normality and adjustment for multiple comparisons
☐	☒	A full description of the statistics including central tendency (e.g. means) or other basic estimates (e.g. regression coefficient) AND variation (e.g. standard deviation) or associated estimates of uncertainty (e.g. confidence intervals)
☐	☒	For null hypothesis testing, the test statistic (e.g. *F*, *t*, *r*) with confidence intervals, effect sizes, degrees of freedom and *P* value noted<br>*Give P values as exact values whenever suitable.*
☒	☐	For Bayesian analysis, information on the choice of priors and Markov chain Monte Carlo settings
☒	☐	For hierarchical and complex designs, identification of the appropriate level for tests and full reporting of outcomes
☐	☒	Estimates of effect sizes (e.g. Cohen's *d*, Pearson's *r*), indicating how they were calculated
☐	☒	Clearly defined error bars<br>*State explicitly what error bars represent (e.g. SD, SE, CI)*

Our web collection on statistics for biologists may be useful.

Software and code

Policy information about availability of computer code

Data collection	The filtered high-quality reads were mapped to the locust reference genome using Burrows-Wheeler Aligner (BWA) with the command 'mem -t 4 -k 32 –M'.<br>For SNP calling: SAMtools mpileup -q 1 -C 50 -t SP -t DP -m 2 -F 0.002 -uf ref.fa -b bam.list | bcftools call -mv -f GQ |vcfutils_latest.pl varFilter -Q 20 -d 2 -D 1500000 - > locust.raw.vcf

Data analysis

Phylogenetic tree: software TreeBestv1.9.2, 'treebest nj -b 100 input.fa >tree.out'
PCA analysis was conducted using EIGENSOFT3.0 and the significance of eigenvectors was determined using the Tracey-Widom test. 'gcta64 --grm locust --pca 3 -out locust'
Population genetic structure: examined via an expectation maximization algorithm, as implemented in the program FRAPPEv1.1. 'frappe structure.param'
Effective population size (Ne): using a hidden Markov model approach as implemented in pairwise sequentially Markovian coalescence with parameter as follows: '–N30 –t15 –r5 and –p '4+25*2+4+6'.
Squared correlation coefficient (r^2): software Haplo View v4.2 63. Parameters in the program were set as '-n –dprime-minMAF 0.05'.
FST: vcftools --gzvcf input.vcf.gz --weir-fst-pop pop1.list --weir-fst-pop pop2.list --fst-window-size 100000 --fst-window-step 50000 --out pop1-pop2
Cross-population composite likelihood approach (XP-CLR): XP-CLR version 1.0 10-30-2009. 'XPCLR -xpclr genofile1 genofile2 mapfile outputFile -w1 0.005 200 2000 chrN -p 0.95'.
Tajima's D: VCFTOOLS with the parameter '–TajimaD'.

For manuscripts utilizing custom algorithms or software that are central to the research but not yet described in published literature, software must be made available to editors/reviewers upon request. We strongly encourage code deposition in a community repository (e.g. GitHub). See the Nature Research guidelines for submitting code & software for further information.

Data

Policy information about availability of data

All manuscripts must include a data availability statement. This statement should provide the following information, where applicable:

- Accession codes, unique identifiers, or web links for publicly available datasets
- A list of figures that have associated raw data
- A description of any restrictions on data availability

The published reference genome of migratory locust used for mapping is available at LocustBase [http://159.226.67.243/download.htm]. Fastq files of the genome sequence for each of 24 locusts are available at BioProject SRP136595 (sample accession no. SRX3855011 - SRX3855034) in NCBI Sequence Read Archive (SRA) [https://www.ncbi.nlm.nih.gov/sra/?term=SRP136595]. Fastq files of the transcriptome sequence for hypoxia treatment are available at SRA BioProject SRP135947 (sample accession no. SRX3808855 - SRX3808866) [https://www.ncbi.nlm.nih.gov/sra/?term=SRP135947]. Fastq files of the transcriptome sequence for gene expression in different tissues are available at SRA BioProject SRP134674 (sample accession no. SRX3779821 - SRX3779826) [https://www.ncbi.nlm.nih.gov/sra/?term=SRP134674]. The GenBank accession number for the mRNA sequence of locust PTP1B is MH973608. A reporting summary for this article is available as a Supplementary Information file.

Field-specific reporting

Please select the best fit for your research. If you are not sure, read the appropriate sections before making your selection.

☐ Life sciences ☐ Behavioural & social sciences ☒ Ecological, evolutionary & environmental sciences

For a reference copy of the document with all sections, see nature.com/authors/policies/ReportingSummary-flat.pdf

Ecological, evolutionary & environmental sciences study design

All studies must disclose on these points even when the disclosure is negative.

Study description

In this study, we have compared the genetic differences between Tibetan and lowland locust populations and investigated the genetic mechanism of hypoxia adaptation for Tibetan locust. For genome sequencing, 12 field collected Tibetan locust samples and 10 field collected lowland locust samples were used. For functional and transcriptome study, experimental groups were treated with 10 kPa hypoxia for 120 h, and normoxia groups were used as control. For in vivo study, each experiment contains at least 4 replicates. For western blot and in vitro experiment at least 3 replicates were used. Each replicate contains three locusts.

Research sample

1) Tibetan locusts, Locusta migratoria populations that habitat in Tibetan plateau; 2) Lowland locusts, Locusta migratoria populations that habitat in South China plain. The Tibetan and South China lowland locust populations belong to same lineage around worldwide locust populations. Both male and female adult locusts were selected randomly for whole genome resequencing.

Sampling strategy

Locusts sampled at each locality were caught at more than ten sites that are spaced over 300 meters between each other. Approximately 50 locusts were collected at each site.

Data collection

For quantitative PCR, data were generated by a LightCycler 480 instrument (Roche, USA). Gene expression level was calculated via 2^-ΔCt, significance level was analyzed by Student's t-test.
For metabolite measurement, trehalose and glucose was tested by Agilent 6890N-5973N, the data were generated by software AMDIS.
For Colorimetric/Fluorometric Assay, data were generated via a multi-mode detection platform (Paradigm, USA) and analysed with software GraphPad Prism 5.
For micrographs, photos were captured by LSM 710 confocal fluorescence microscope (Zeiss, Germany). At least five tissue sections (five individuals) from each treatment were used for data analysis.
For lifespan assay, 30 locusts was used in each group. Death events was calculated twice a day. The final data was analysed with software SPSS.
Data were collected by Ding Ding.

Timing and spatial scale	For whole genome re-sequencing, locust samples from Maizhikungga were collected in 2005, locust samples from Lhasa were collected in 2014, Two samples from Shanan and Doilong were collected in 2015, another sample from Doilong was collected in 2005 and samples from lowland were collected in 2014. For functional study, Tibetan locust samples were collected from Lhasa in 2014, 2015 and 2016.
Data exclusions	No data was excluded.
Reproducibility	In the current study, quantitative experiments were performed with at least three biological samples. Some important assays were repeated for 3 times for each sample. All attempts to repeat the experiment were successful.
Randomization	Samples for assay were randomly allocated.
Blinding	Blinding was used for all data acquisition and analysis.

Did the study involve field work? ☐ Yes ☒ No

Reporting for specific materials, systems and methods

Materials & experimental systems

n/a	Involved in the study	
☒	☐	Unique biological materials
☐	☒	Antibodies
☐	☒	Eukaryotic cell lines
☒	☐	Palaeontology
☐	☒	Animals and other organisms
☒	☐	Human research participants

Methods

n/a	Involved in the study	
☒	☐	ChIP-seq
☒	☐	Flow cytometry
☒	☐	MRI-based neuroimaging

Antibodies

Antibodies used	phosphorylation antibodies of InR (1:2,000; Abcam, ab62321, USA) and AKT (1:1,000; CST, 4054S, USA); antibody of locust PTP1B are custom-made and purified by Abclonal Company(China).
Validation	For phosphorylation antibodies of InR (Abcam, ab62321, USA), Kong Q et al., 2014. This antibody specifically recognize Y1185 of human InR which is conserved in human and locust.<br>For phosphorylation antibodies of AKT (CST, 4054S, USA), Dionne et al., 2006. This antibody specifically recognize Ser505 in Drosophila AKT which is conserved in Drosophila and locust.<br>For locust PTP1B antibody. This antibody is made by Abclonal Company (China). The immunogen of this antibody is full length locust PTP1B recombinant protein. This antibody was validated by RNAi assay in this study (Fig.4e).

Eukaryotic cell lines

Policy information about cell lines

Cell line source(s)	The S2 cell line that used in this study was bought from Thermo Fisher Scientific.
Authentication	The S2 cell line was not authenticated.
Mycoplasma contamination	Not tested for mycoplasma contamination
Commonly misidentified lines (See ICLAC register)	No commonly misidentified cell lines were used in this study.

Animals and other organisms

Policy information about studies involving animals; ARRIVE guidelines recommended for reporting animal research

Laboratory animals	*For laboratory animals, report species, strain, sex and age OR state that the study did not involve laboratory animals.*
Wild animals	*Provide details on animals observed in or captured in the field; report species, sex and age where possible. Describe how animals were caught and transported and what happened to captive animals after the study (if killed, explain why and describe method; if released, say where and when) OR state that the study did not involve wild animals.*
Field-collected samples	Field-collected locusts were maintained in the laboratory at the Institute of Zoology, Chinese Academy of Sciences in Beijing (<50

Field-collected samples	m). All locusts were reared in ventilated cages (50 × 50 × 50 cm) at a density of approximately 150 individuals per cage and fed with fresh wheat seedlings and wheat bran. The culturing environment was kept constant with a 14 light (L):10 h dark (D) photo regime at 28 ± 1 °C. The cultures were maintained in the laboratory for at least two generations prior to the experiments. The 7 d-old male adults were collected for assays.
